# Identification of key gene networks controlling organic acid and sugar metabolism during star fruit (*Averrhoa carambola*) development

**DOI:** 10.1186/s12870-024-05621-4

**Published:** 2024-10-10

**Authors:** Xinyu Xu, Lianhuan Xu, Zirui Yang, Lei Chen, Yiqing Wang, Hui Ren, Zehuang Zhang, Yousry A. El-Kassaby, Shasha Wu

**Affiliations:** 1 The Innovation and Application Engineering Technology Research Center of Ornamental Plant Germplasm Resources in Fujian Province, National Long term Scientific Research Base for Fujian Orchid Conservation, Fujian Agriculture and Forestry University, Fuzhou, 350002 China; 2 Department of Forest and Conservation Sciences, Faculty of Forestry, The University of British Columbia, Vancouver, BC Canada; 3 Horticulture Research Institute, Guangxi Academy of Agricultural Sciences, Nanning, 530007 China; 4 Fruit Research Institute, Fujian Academy of Agricultural Sciences, Fuzhou, 350003 China

**Keywords:** *Averrhoa carambola* ‘Beiliusuan No. 1’, Sugar and acid metabolism, Metabolome, Transcriptome, Genome

## Abstract

The sugar and organic acid content significantly impacts the flavor quality of star fruit, and it undergoes dynamic changes during development. However, the metabolic network and molecular mechanisms governing the formation of sugar and organic acid in star fruit remain unclear. In this study, 23 of 743 components were detected by metabonomic analysis. The highest metabolites contents were organic acids and derivatives. The highest sugar content in the fruit was fructose and glucose, followed by sucrose, which proved that *A. carambola* is a hexose accumulation type fruit. Genome identification preliminarily screened 141 genes related to glucose metabolism and 67 genes related to acid metabolism. A total of 7,881 unigenes were found in transcriptome data, 6,124 differentially expressed genes were screened, with more up-regulated than down-regulated genes. Transcriptome and metabolome association analysis screened seven core candidate genes related to glucose metabolism and 17 core genes highly related to organic acid pathway, and eight differentially expressed sugar and acid genes were selected for qRT-PCR verification. In addition, 29 bHLHs and eight bZIPs transcription factors were predicted in the glucose metabolism pathway, and 23 MYBs, nine C2H2s transcription factors and one GRAS transcription factor was predicted in the acid metabolism pathway, and transcription factors have both positive and negative regulatory effects on sugar and acid structure genes. This study increased our understanding of *A. carambola* fruit flavor and provided basic information for further exploring the ornamental and edible values of star fruit.

**Supplementary Information:**

The online version contains supplementary material available at 10.1186/s12870-024-05621-4.

## Introduction

Star fruit (*Averrhoa carambola*), belonging to the genus Averrhoa and the family Oxalidaceae, is one of the main fruit trees in tropical and subtropical areas [1]. The fruit originated from South-East Asia, but is commonly harvested in various other Asian countries, South America, the United States and Oceania [2]. Because the cross section of *A. carambola*'s fruit is star-shaped, it is also called “star pear”. Its ripe fruit has a sweet, juicy, slightly sour tastes with a pleasant aroma. Additionally, the fruit has important medicinal and edible values. Continuous selection and improvement efforts of star fruit resulted in creating a prominent position in the fruit consumption market; however, there remains potential for enhancing its fruit flavor and quality compared to better compete with other popular fruits. The flavor and quality of star fruit are primarily driven by factors such as sugar and organic acid contents, which are significant metabolites playing a crucial role in determining its fruit quality [3]. However, the molecular mechanism governing the sugar and organic acid pathways and metabolism of star fruit have yet to be explored.

Sugar serves as the fundamental raw material for the formation of fruit quality and flavor substances, as well as the synthesis of other nutrients. Therefore, the sugar content plays a crucial role as an important determinant of fruit quality [4]. Recent studies have highlighted the significant role of transport and metabolism of photoassimilates in fruit sugar accumulation at the physiological, molecular, and signal transduction levels. Additionally, fructose, glucose, and sucrose serve as crucial signal molecules of soluble sugar, playing important roles in regulating plant metabolic process and defense response mechanism [5, 6]. Sucrose phosphate synthase (SPS), sucrose synthase (SS), invertase (INV), and hexokinase (including glucokinase and fructokinase) play major roles in this metabolic process [7–9]. Recent studies have identified these enzymes have been found in *Saccharum officinarum*, *Cucumis sativus*, and *Cucumis melo*. Additionally, sugar transporters have been found to mediate the process of sugar accumulation [10–13]. A large number of sugar transporter gene families, such as MST, SUT and SWEET families, have been identified in the model plant *Arabidopsis thaliana* [14–16]. A total of 58 and 49 members of MST family were identified in the non-model plants *Vitis vinifera* and *Lycopersicon esculentum*, respectively [17, 18]. The sucrose transporter SUT belongs to a small gene family, with only four family members identified in grape and *Oryza sativa* [19, 20]. The SWEET family has been reported in *Glycine max*, *Gossypium hirsutum*, and *Brassica napus* [21–23]. Organic acids are organic compounds with carboxyl structure, present in plant fruits, seeds and leaves. Variation in the composition and content of organic acids influences the distinctive flavors of fruits. Flavor quality constitutes an important part of fruit quality formation [24].

In fruits, mitochondria serve as the primary site for organic acid metabolism, encompassing the tricarboxylic acid (TCA) cycle within mitochondria and the transport of malic acid, citric acid, and isocitrate into vacuoles [25]. Previous studies on the composition and content changes of organic acids in fruits have indicated that citric acid predominates in muskmelon, followed by malic, oxalic, and succinic acids, among others. Apple (*Malus pumila*) fruit contains 16 organic acids, with malic acid accounts for 84% [26]. The main organic acid components of ‘Furong’ plum fruit across developmental stages were malic acid (73.83–92.10%), followed by tartaric acid (4.59–14.26%), low contents of citric, oxalic, acetic and succinic acids (0.47-7.21%), and trace amount of fumaric acid [27]. In the entire pathway of organic acid metabolism, certain genes, such as malate dehydrogenase (MDH), malic enzyme (ME), citrate synthase (CS), phosphoenolpyruvate carboxylase (PEPC), and organic acid transport, play crucial roles in the formation of organic acids [28, 29]. After apple fruit harvest, proper calcium treatment significantly increased the expression of *MdMDH1*, *MdMDH2*, *MdPEPC1*, and *MdPEPC2* [30]. In *Ficus carica*, after pollination and during fruit development, citrate synthase and phosphoenolpyruvate carboxylase increase, affecting fruit quality improvement [31].

Recently, transcriptome and metabolome data association analyses have become effective technical means for exploring the function of new genes and clarifying the regulatory network of metabolic pathways [32, 33]. These analyses have been widely used in studying muskmelon fruit metabolism [34], kiwifruit (*Actinidia chinensis*) fruit flavor [35], and *Ficus carica* fruit flavor and flavonoid synthesis [36]. In watermelon (*Citrullus lanatus*), Umer et al. [37] used transcriptome and metabolic group to construct the co-expression patterns of gene networks related to sugar and organic acid metabolism. They identified three gene networks modules, comprising 243 genes related to sugar and organic acids, and identified seven genes involved in sugar and organic acid metabolism. Wu et al. [38] identified 15 structural genes and 12 transcription factors involved in the biosynthesis, transport, and regulation of fruit wax through the co-expression network analysis of differential metabolites and genes. This elucidated the molecular mechanism of pear (*Pyrus* spp.) fruit wax formation. Wang et al. [39] integrated metabolic grouping and genome-wide transcriptome analysis comprehensively, revealing the regulatory network of ‘Hongyang’ kiwifruit flavor formation. This, enabled the identification of key structural genes and transcriptional factors regulating the metabolism of soluble sugar, organic acids, and important volatiles in kiwifruit. It should be noted that the genes and metabolic pathways causing poor flavor in star fruit have not been reported. In the breeding process, two types of star fruit varieties have been selected: one with a sour taste and the other with sweet taste. Currently, there is limited research on the varieties with sour flavor, and the reasons for the poor flavor of star fruit and the molecular mechanism of regulating star fruit flavor remain unclear. In this study, multi-combinatorial analysis was used to mine the candidate genes related to sugar and organic acid metabolism in *A. carambola* fruit and construct transcriptional regulatory network. This represents the first comprehensive investigation into the molecular mechanisms underlying the development of sour and sweet flavors in *A. carambola*. The study aimed to provide data sources for the functional verification of related genes. Ultimately, this work will clarify the molecular mechanism of fruit flavor metabolism of *A. carambola*, and offer new perspectives for the cultivation and promotion of new varieties of dual-purpose star fruit plants.

## Results

### Star fruit development and sugar and organic acid content dynamic changes

**Fig. 1:**
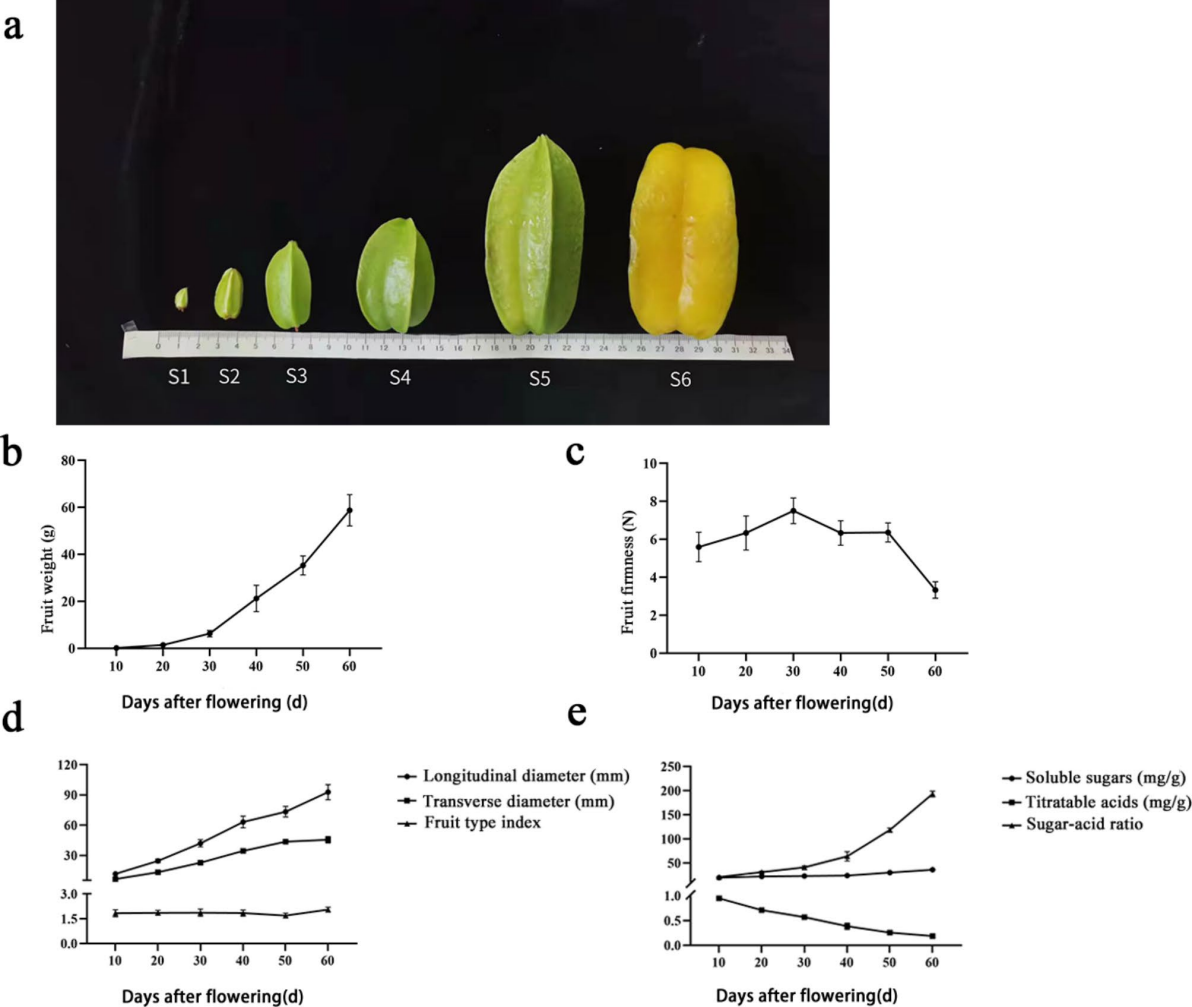
*A. caramhola* fruit morphology and physiological indexes development. **a** Star fruit at different developmental stages (collection stages of fruit samples: S1 (Green fruit stage), S2 (Young fruit stage), S3 (Stigma fruit stage), S4 (Hard fruit stage), S5 (Veraison fruit stage), and S6 (Mature fruit stage)). **b** Fruit weight after flowering. **c** Fruit firmness after flowering. **d** Fruit longitudinal and transverse diameter after flowering. **e** Fruit soluble sugars and titratable acids after flowering

We investigated the dynamic changes of sugar and organic acid content during the star fruit 60 days development (Fig. 1a). The accumulation and growth of fruit quality basically accorded with the single ‘S’ growth curve. As fruit development progressed, we speculated that the observed increase in single fruit weight may be attributable to fruit moisture content increase. This surge occurred notably around 30 days after flowering, signifying this period as pivotal in fruit development (Fig. 1b). Fruit firmness increased at first and then decreased (Fig. 1c) with gradual increase in growth and development between 10 and 40 days after flowering, then rapid decrease from 40 days after flowering to the ripening stage, followed by the start of fruit softening. Fruit transverse and longitudinal diameters showed continuous growth trend during the whole development period (Fig. 1d), with overall relative growth rate of the longitudinal diameter that was higher than that of the transverse diameter. Between 50 and 60 days after flowering, the longitudinal diameter increased rapidly, while the transverse diameter showed minor increase., with fruit shape index (longitudinal diameter / transverse diameter) increase between 50 and 60 days. During fruit development, total soluble sugar content increased slowly, while the titratable acid decreased rapidly, and the ratio of sugar to acid increased significantly in the middle and later stage of fruit development (Fig. 1e).

### Analysis of sugar and organic acid metabolism during star fruit development

**Fig. 2:**
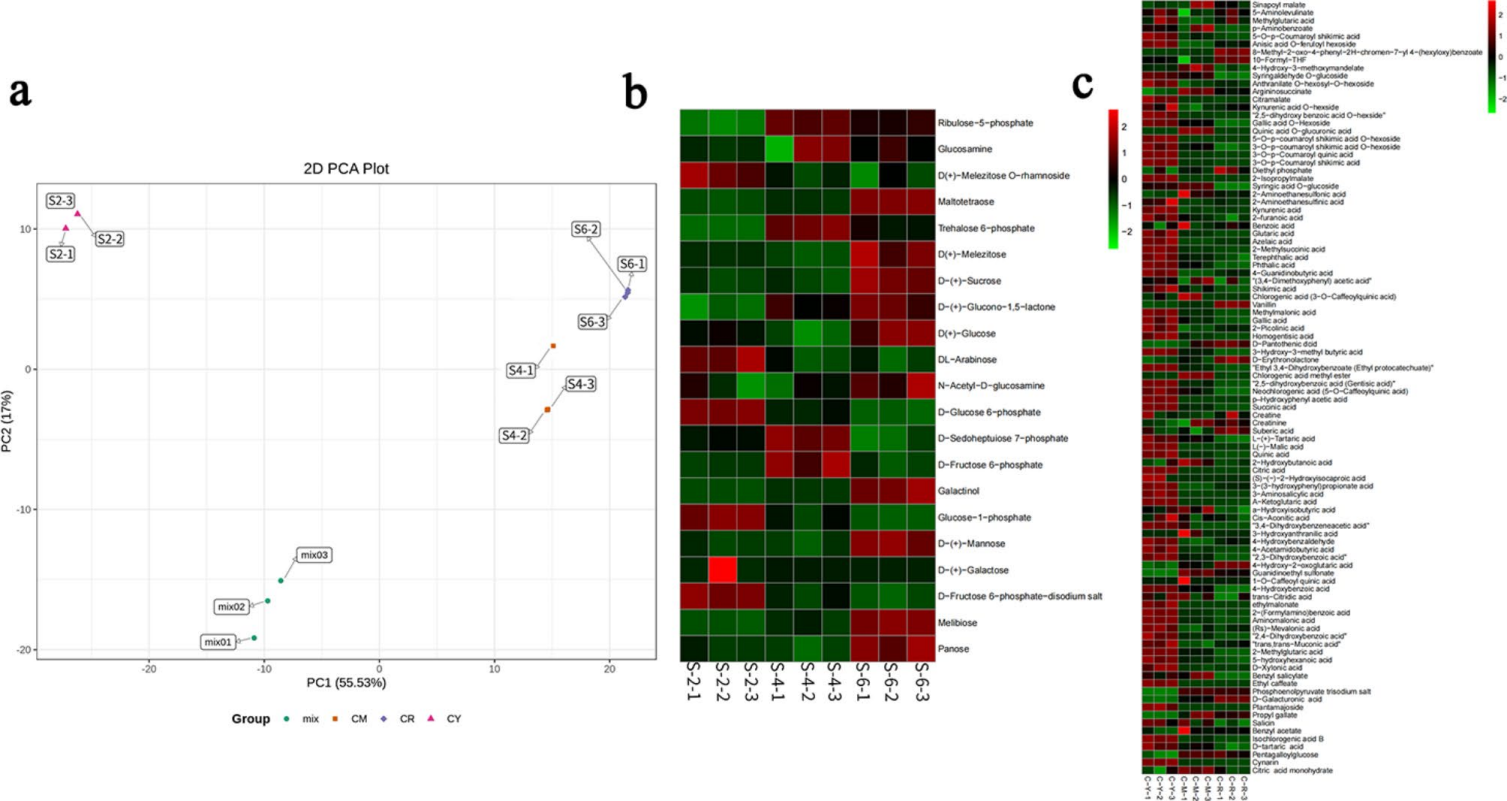
Principal component analysis and heat map differential metabolites of sugar and organic acid in star fruit at different development stages. **a** Principal component analysis (PCA). **b** Sugar and organic acid contents at different development stages. **c** Organic acid contents at different development stages

For metabolism analysis, correlation analysis showed that sample repeatability was good in S2, S4 and S6 sample groups during fruit development (Fig. S1). PCA analysis of S2, S4, S6 and quality control samples showed significant separation of PC1 among S2, S4 and S6, indicating significant changes in metabolites among samples at different developmental stages (Fig. 2a). A total of 22 sugar contents were identified (Table S1), among which D-fructose 6-phosphate-disodium salt was the highest, accounting for 19.76% of the total carbohydrate metabolites, followed by glucose-1-phosphate (19.57%). Galactinol dihydrate content was the lowest. The top five carbohydrate metabolites were D-fructose 6-phosphate-disodium salt (19.76%), glucose-1-phosphate (19.57%), D-fructose 6-phosphate (19.26%), D-(+)-sucrose (12.75%), and melibiose (10.09%). Eight carbohydrate differential metabolites were screened in the S2 vs. S4, S2 vs. S6, S4 vs. S6, which showed an up-regulation trend in the three developmental stages. The contents of 23 carbohydrate metabolites were significantly different among the different stages, and they were mostly carbohydrate metabolites in fruit development and mature fruit stage (S6) and were higher than those in young fruit stage (S2) (Fig. 2b). Among them, D(+)-melezitose O-rhamnoside, D-glucose-6-phosphate, glucose-1-phosphate, and D-fructose 6-phosphate-disodium salt contents gradually decreased with fruit development, and peaked in the young fruit stage (S2). Glucosamine, ribulose-5-phosphate, trehalose 6-phosphate, D-sedoheptuiose 7-phosphate, and D-fructose-6-phosphate contents were the highest in the hard fruit stage (S4) and began to decrease with fruit ripening (S6). Maltotetraose, D(+)-melezitose, D-(+)-sucrose, D-(+)-glucono-1,5-lactone, D(+)-glucose, D-(+)-Mannose, melibiose, and panose contents in the fruit ripening stage (S6) were higher than those in the other two stages (S2 and S4). Glucose and fructose accumulated at the beginning of fruit development and decreased during ripening, while sucrose accumulated during fruit ripening. We screened 10 carbohydrate differential metabolites in S2 vs. S4, S4 vs. S6, and S2 vs. S6, and no down-regulated differential metabolites were found, indicating that sugars were differentially up-regulated during fruit development (Table 1).

On the other hand, 98 organic acids and derivatives were identified from star fruit (Table S2), among which the content of quinic acid was the highest, accounting for 32.98% of the total metabolite content, followed by methyl malonic acid (11.84%). Quinic acid O-glucuronic acid content was the lowest, accounting for only 0.000785% of the total metabolite content while the top 10 metabolites were quinic acid (32.98%), methylmalonic acid (11.84%), succinic acid (11.48%), citric acid (5.45%), 10-formyl-THF (4.81%), p–hydroxyphenyl acetic acid (3.75%), 2,5-dihydroxy benzoic acid O-hexside (3.40%), D-galacturonic acid (2.39%), D-xylonic acid (2.16%), and terephthalic acid (1.79%). The expression of 98 organic acid metabolites were analyzed (Fig. 2c) and most of the organic acids and derivatives were highly expressed in the early stage of fruit development, and decreased gradually with the fruit development and ripening. Among them, nine organic acids were highly expressed in the young fruit stage (8-methyl-2-oxo-4-phenyl-2 H-chromen-7-yl 4-(hexyloxy) benzoate, 10-formyl-THF, diethyl phosphate, vanillin, D-pantothenic acid, D-erythronolactone, suberic acid, 4-hydroxy-2-oxoglutaric acid, and D-galacturonic acid). A total of 18 organic acids were highly expressed in the hard fruit stage (S4), while the rest were highly expressed in the young fruit stage (S2). We screened 53 organic acid differential metabolites in S2 vs. S4, including eight up- and 45 down-regulated; 26 organic acid differential metabolites in S4 vs. S6, including six up- and 20 down-regulated; and 56 organic acid differential metabolites in S2 vs. S6, of which 8 were up- and 48 down-regulated. For organic acids, the down-regulation of differential metabolites was higher than that of up-regulation, indicating that the former plays an important role in fruit development than the latter (Table 1).

**Table 1:** Analysis of differences of carbohydrate and organic acid metabolites in different periods

Material category	Metabolites up-down-regulated	The number of DEGs		
		S2-S4	S4-S6	S2-S6
Carbohydrates	Up-regulated Down-regulated	2 0	2 0	6 0
Organic acids and derivatives	Up-regulated Down-regulated	8 45	6 20	8 48

### Identification of sugar and organic acid related genes in the star fruit genome

**Fig. 3:**
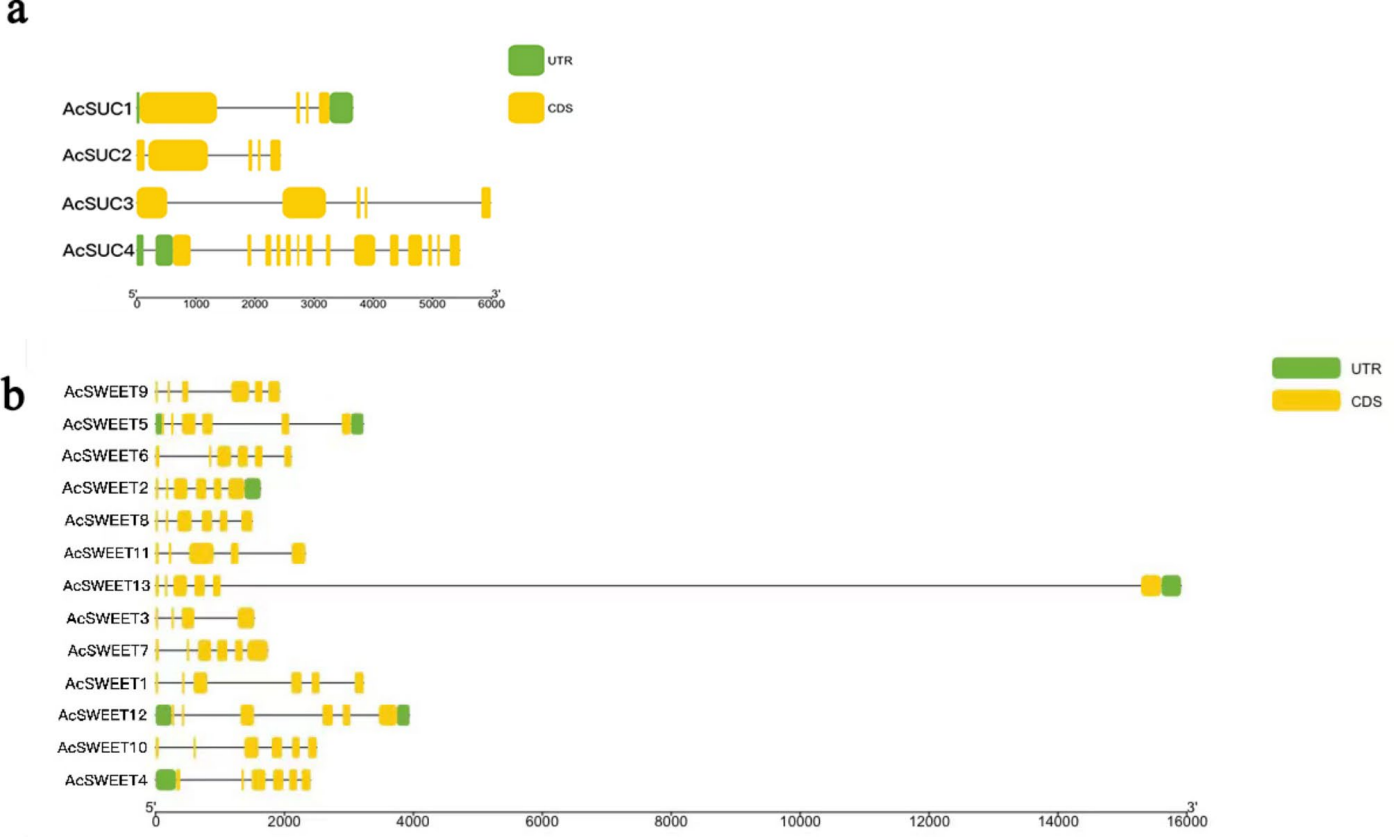
Gene structure model of sucrose-related gene families. **a** SUC gene family. **b** SWEET gene family

Four sucrose phosphate synthases (SPS), seven soluble sugars (SS), 21 invertase (INV), three hexokinases (HXK), and 15 fructokinases (FRK) genes were identified from the *A. carambola* genome database by Blast comparison (Table S3), 91 sugar transporter genes were obtained (Table S4), which were divided into four sucrose (SUC), 13 sweet sugars (SWEET), and 74 monosaccharide general transporters genes (MFS). Three *AcSUCs* were identified to belong to the sucrose transporter (SUC) family in *A. carambola*. We speculate that the gene structure model of *AcSUC1* had four exons and two introns (Fig. 3a), *AcSUC2* and *AcSUC3* had five exons, but *AcSUC4* contained 14 exons. The phylogenetic tree showed that four *AcSUCs* were classified into three different branches, and four *AcSUCs* were highly similar to four strawberry sucrose transporters, indicating that they were closely related (Fig. S2). In addition, seven *AtSUCs* genes clustered in one branch, which may be overactive. The gene structure model showed that the *AcSWEETs* transporter contained two introns and four exons, and only four *AcSWEETs* members contained introns (Fig. 3b). Most *AcSWEETs* contained six exons, and the length of exons at 3’ end is longer than that at 5’ end. The phylogenetic analysis showed that *AcSWEETs* and *AtSWEETs* are clustered into four subbranches (Fig. S2). Most *AcSWEETs* members have high similarity with *AtSWEETs* family members, and *AcSWEET3* is separated from the other genes.

A total of 72 acid metabolism related genes were identified from the genome of *A. carambola* (Table S2b). These included four aconitase (ACO), two NADP-Isocitrate dehydrogenase (NADP-IDH), four NAD-Isocitrate dehydrogenase (NAD-IDH), five NADP-mailc enzyme (NADP-ME), four glutamine synthetase (GS), NAD-malate dehydrogenase (NAD-MDH), three phosphoenolpyruvate carboxylase (PEPC), and seven Glyceraldehyde-3-phosphate dehydrogenases (GADPH) genes. Additionally, four citrate synthase (CS), four glutamate dehydrogenase (GDH), five glutamate decarboxylase (GAD), one phosphoenolpyruvate carboxykinases (PEPCK), 12 aluminum-activated malate transporters (ALMT), three V-ATPase, and four membrane-bound proton-pumping pyrophosphate (V-Ppase) genes were also identified.

### Transcriptome analysis and differentially expressed genes

After quality filtering, the *A. carambola* transcriptome sequencing resulted in about 5.7Gb clean reads, and the clean bases produced an average amount of 8.6 Gb data. The quality values Q20 and Q30 were 97.42 and 92.93%, respectively, and the average GC content was 44.56%, meeting the assembly requirements of subsequent sequences (Table 2). All full-length transcript sequences were functionally annotated and compared with NR, GO, KEGG, Swiss-Port, and KOG5 databases (Table S3). A total of 7,751 (98.3%), 5,477 (69.5%), 3,072 (38.9%), 5,580 (71.7%), and 7,247 (92.0%) transcript sequences were annotated to these five databases, respectively, with 2,590 (33.3%) common across the five databases.

**Table 2:** Statistics of RNA-seq products in four developmental stages

Sample	Clean reads	Cleans bases	Q20(%)	Q30(%)	GC(%)
S-2-1	63,115,132	94,672,698	97.49	93.03	44.43
S-2-2	56,145,702	84,218,553	97.47	93.04	44.78
S-2-3	63,289,202	94,933,803	97.53	93.18	44.55
S-4-1	50,547,268	75,820,902	97.4	92.92	44.67
S-4-2	53,161,454	79,742,181	97.41	92.92	44.60
S-4-3	50,535,844	75,803,766	97.36	92.77	44.69
S-6-1	61,373,698	92,060,547	97.32	92.75	44.19
S-6-2	63,221,398	94,832,097	97.49	93.09	44.56
S-6-3	58,569,980	87,854,970	97.32	92.69	44.55

**Fig. 4:**
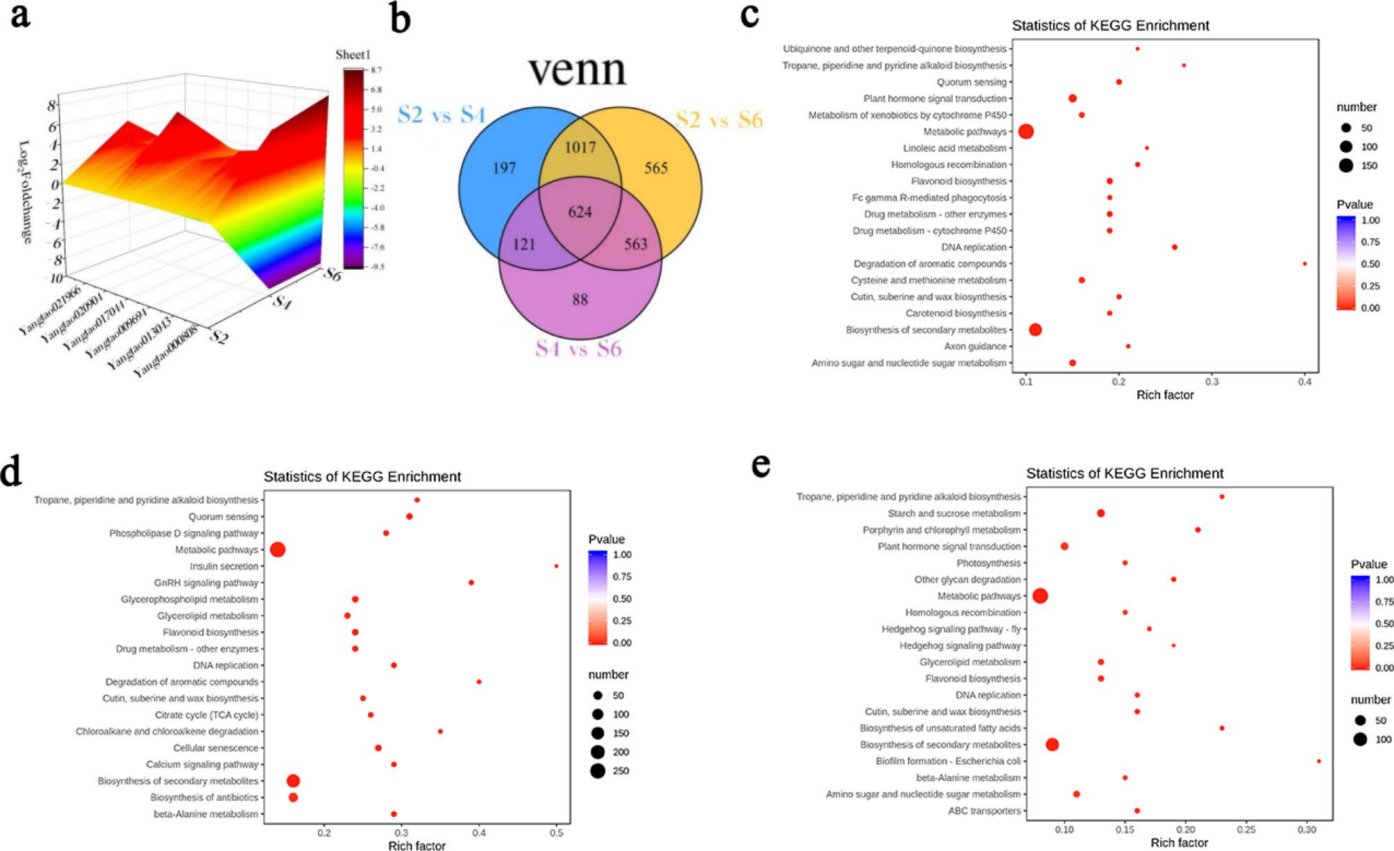
Transcriptome analysis of DEGs in three comparative groups. **a** For the expression patterns of all differentially expressed genes, the blue to red color bars indicates log2FPKM ranging from − 9.5 to 8.7. **b** Venn diagrams of differentially expressed genes. **c** KEGG classification of differentially expressed genes in S2 vs. S4. **d** KEGG classification of differentially expressed genes in S4 vs. S6. e KEGG classification of differentially expressed genes in S2 vs. S6

Additionally, a total of 6,124 differentially expressed genes (DEG) were obtained. According to S2 vs. S4, S4 vs. S6, and S2 vs. S6 the DEG were divided into three comparison groups, and the number of DEG between groups was counted. The results showed that there were 1,959, 1,396, and 2,769 DEG between S2 and S4, S4 and S6, and S2 and S6, respectively (Table 3). The log2FPKM of these genes is represented by three-dimensional graph (Fig. 4a), there are significant differences in the log2FPKM value of many differential genes between S4 and S6. The total DEGs is divided into four groups according to different expression patterns. These groups contained either up- (group I 537), down- (group II 1,571 down), up- followed by down- (group III 175), and down- followed by up-regulated genes (group IV 179) and the three comparison groups intersected (Fig. 4b). The DEGs in the three groups were compared with the KEGG database, and the first 20 metabolic pathways with the most significant differences are displayed and analyzed (Fig. 4c). During the process of fruit development, DEGs were enriched in metabolic pathway (ko01100), biosynthesis of secondary metabolites (ko01110), biosynthesis of antibiotics (ko01130), and plant hormone signal transduction (ko04075). In addition, starch and sucrose (ko00500) and carbon metabolism (ko01200), phenylpropanoid biosynthesis (ko00940), biosynthesis of amino acids (ko01230), microbial metabolism in diverse environments (ko01120), amino sugar and nucleotide sugar metabolism (ko00520), flavonoid biosynthesis (ko00941), among other metabolic pathways involved in the fruit development process. Comparing of young (S2) with hard fruit (S4) (Fig. 4c), 188 DEGs and 82 differential metabolites (DEMs) were enriched into metabolic pathway (ko01100), 118 DEGs and 57 DEMs were enriched into biosynthesis of secondary metabolites pathway (ko01110), 34 DEGs and 19 DEMs were enriched into microbial metabolism in diverse environments pathway (ko01120), 34 DEGs and 21 DEMs were enriched into biosynthesis of antibiotics pathway (ko01130), 21 DEGs and 11 DEMs were enriched into phenylpropanol biosynthesis pathway (ko00940) (Table S5). Comparison of hard fruit stage (S4) and mature stage (S6) (Fig. 4d), 143 DEGs and 34 DEMs were enriched into metabolic pathways (ko01100), 92 DEGs and 34 DEMs were enriched into biosynthesis of secondary metabolites pathway (ko01110), 25 DEGs and 18 DEMs were enriched into biosynthesis of antibiotics pathway (ko01130), 18 DEGs and 13 DEMs were enriched into microbial metabolism in diverse environments pathway (ko01120), 15 DEGs and 4 DEMs were enriched in the carbon metabolism pathway (ko01200) (Table S6). Comparison between young fruit (S2) and mature fruit (S6) (Fig. 4e), 252 DEGs and 93 DEMs were enriched into metabolic pathway (ko01100), 173 DEGs and 67 differential metabolites were enriched into biosynthesis of secondary metabolites pathway (ko01110), 60 DEGs and 22 DEMs were enriched into biosynthesis of antibiotics pathway (ko01130), 51 DEGs and 24 DEMs were enriched into microbial metabolic pathways in different environments (ko01120), The above metabolic process of 30 DEGs and 15 DEMs enriched to biosynthesis of amino acids pathway (ko01230) is of great significance for the development of fruit and the formation of flavor quality (Table S7).

**Table 3:** The number of different genes in three periods

DEG Set	ALL DEG	Down-regulated	Up-regulated
S2-S4	1,959	1,357	602
S4-S6	1,396	947	449
S2-S6	2,769	1901	868

### Combined analysis of transcriptome and metabolome

In order to further explore the correlation between gene expression and metabolites in star fruit, principal component analysis of DEGs and DEMs showed that the samples clustered with little dispersion. The nine samples were divided into three groups by PCA1 (54.87%) and PCA1 (58.96%). There was little difference among the three biological repeats in the same period (Fig. S3). The results show that the repeatability of the sample is high, and there are great differences between different periods, so it can be used for follow-up result analysis.

**Fig. 5:**
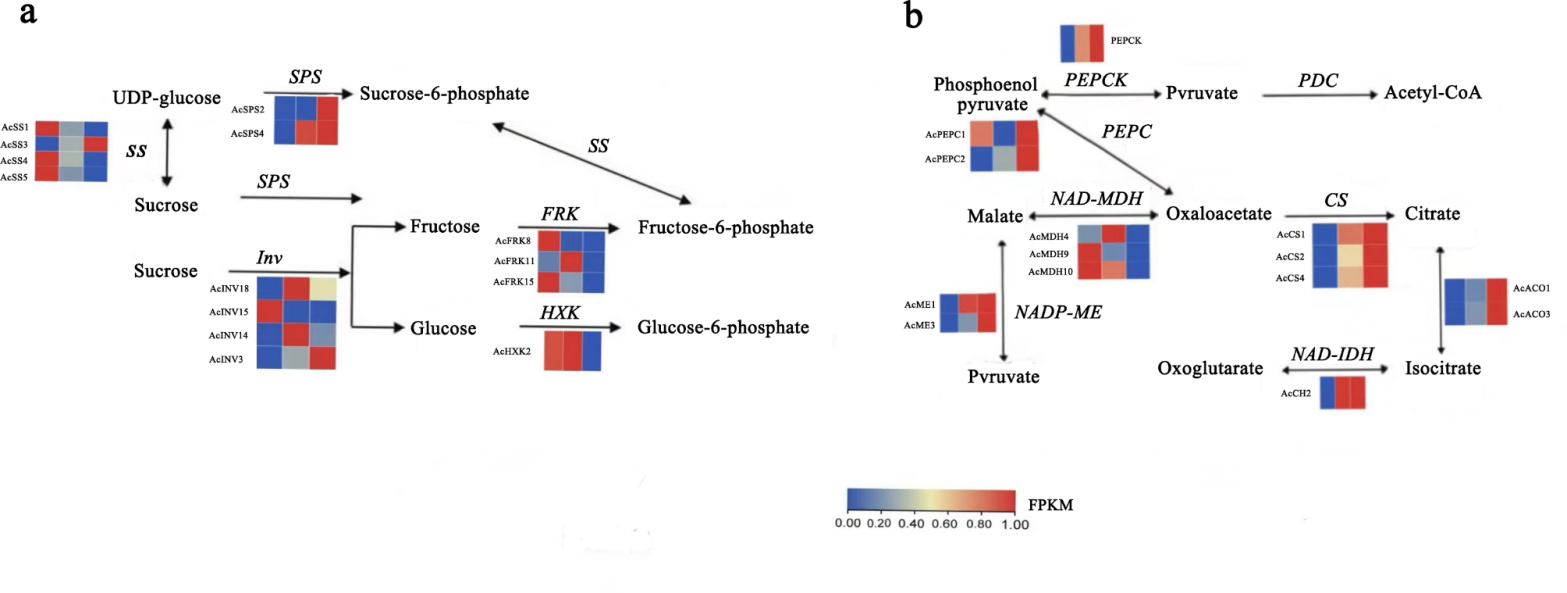
Expression profiles of DEGs involved in the sugar and organic acid biosynthesis. **a** DEGs involved in the sugar acid biosynthesis. **b** DEGs involved in the organic acid biosynthesis. The expression level of each Unigene was represented by three grids, the leftmost grid representing gene expression level in young fruit stage, the middle grid representing gene expression level in hard fruit stage, and the rightmost grid representing gene expression level in mature stage

The young and hard fruit stages, hard and mature fruit stages, young and mature fruit stages were compared, and the correlation between sugar and acid DEGs and DEMs in different fruit stages was analyzed (Fig. S4), and the co-expression network was constructed. Compared with the hard fruit stage (S4), six carbohydrate synthesis related genes and two metabolites were co-expressed in the young fruit stage (S2), in which *AcFRK8* and *AcFRK15* were negatively correlated with maltose and trehalose-6 phosphate, while *AcINV3*, *AcINV14*, *AcSS2*, and *AcSPS4* were positively correlated with the two metabolites. In the pathway of organic acid synthesis, 10 DEGs (*AcALMT11*,* AcPEPC1*,* AcME3*,* AcMDH4*,* AcGAD1*,* AcMDH10*,* AcGADPH5*,* AcCS1*, and *AcCS4*) were co-expressed with 53 DEMs, and the same genes had both positive and negative regulation on different metabolites, among which *AcGADPH5* was only positively correlated with pantothenic acid, and *AcCS4* was separated from other genes. Compared with mature fruit stage, *AcSS4*, *AcFRK15*, *AcINV3*, *AcSS2*, and panose, D (+)-melezitose, D (+)-sucrose, D (+)-galactose, melibiose and maltotetraose were co-expressed in the hard and mature fruit stage. Among them, *AcINV3* was negatively correlated with six sugar metabolites, while other genes were positively correlated with metabolites. Six acid biosynthesis related genes (*AcACO1*,* AcME3*,* AcPEPC1*,* AcMDH4*,* AcACO3*, and *AcVHP1*) were detected in the acid metabolism pathway, which had obvious regulatory networks with 26 DEMs, and most acid biosynthesis genes were negatively correlated with the expression of metabolites, among which folate metabolites were separated from other metabolites. From young to fruit ripening, 6 related DEGs and 6 DEMs (D (+)-melezitose, D (+)-sucrose, D (+)-galactose, melibiose and maltotetraose) were screened. *AcINV3* and *AcSPS4* were negatively correlated with six metabolites, while *AcSS2*, *AcSS4*, *AcFRK8* and *AcFRK15* were positively correlated with metabolites. A total of 57 DEMs were co-expressed with 14 acid synthesis related genes (*AcACO1*,* AcALMT11*,* AcME3*,* AcACO3*,* AcGAD1*,* AcGAD2*,* AcMDH10*,* AcMDH9*,* AcVHP1*,* AcALMT7*,* AcPIDH1*,* AcCS2*,* AcCS1*, and *AcCS4*) in acid metabolism pathways, indicating that acid metabolism pathways play an important role in fruit development and maturation (Fig. S4). Based on the above results, combined with previously related pathway studies, four *AcSSs*, four *AcINVs*, two *AcSPSs*, three *AcFRKs*, and one *AcHXK* DEGs were selected from sugar synthesis genes to predict glucose metabolism pathway (Fig. 5a and b).

### Regulation of transcription factors on sugar and acid metabolism in fruit

**Fig. 6:**
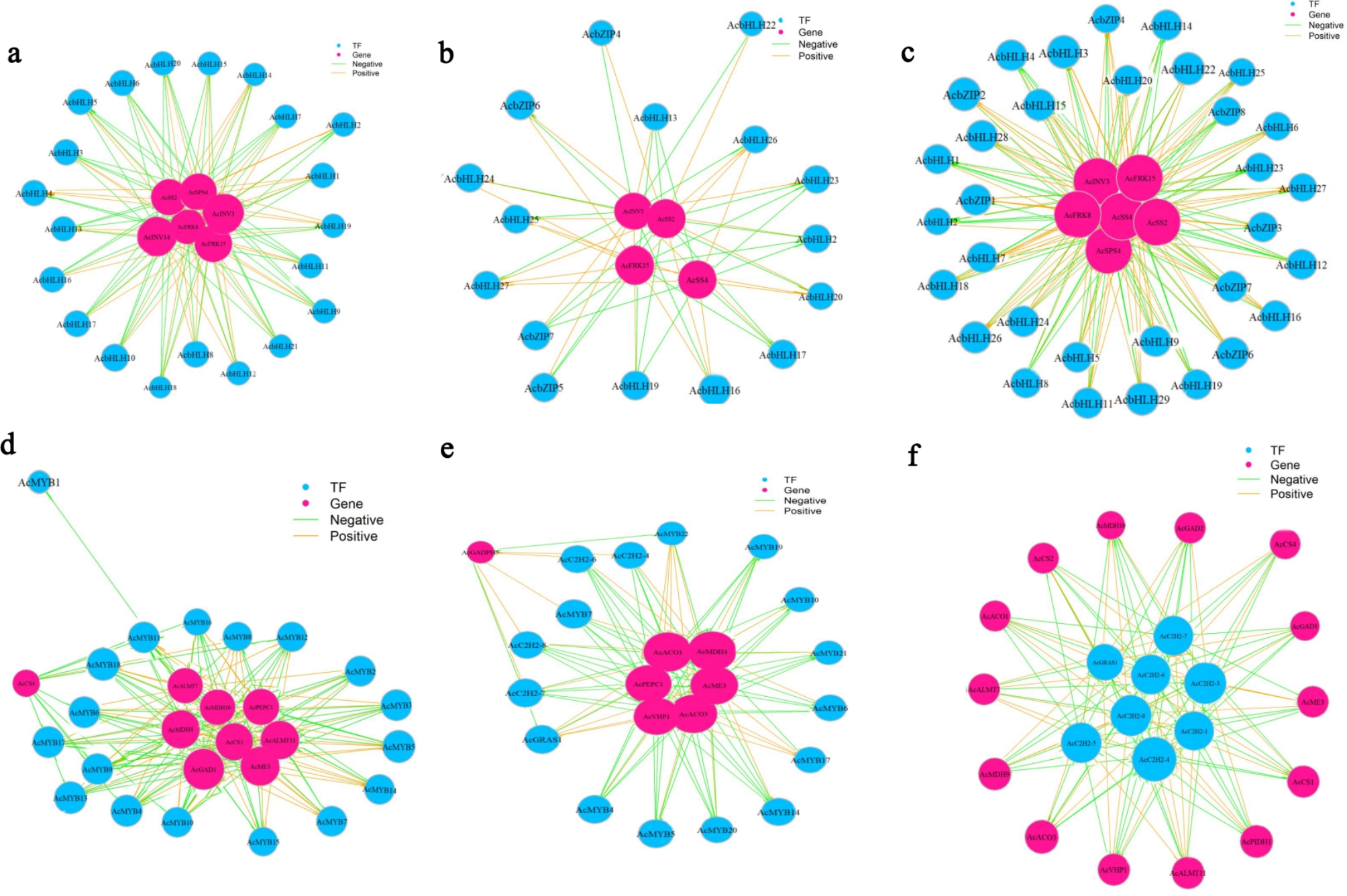
Network diagram of interaction between transcription factors and sugar and organic acid structure genes.**a** young fruit stage vs. hard fruit stage. **b** hard fruit stage vs. mature fruit stage. **c** young fruit stage vs. mature fruit stage. **d** young fruit stage vs. hard fruit stage. **e** hard fruit stage vs. mature fruit stage. **f** young fruit stage vs. mature fruit stage

The transcriptional expression of genes involved in glucose metabolism pathway is regulated by bHLH and bZIP transcription factors. In this study, 29 bHLHs and 8 bZIPs transcription factors were found in the entire fruit development stage of *A. carambola* (Table S8). A total of 21 *AcbHLHs* genes were annotated from young (S2) to mature (S6) fruit stage (Fig. 6a), 12 *AcbHLHs* and 4 *AcbZIPs* transcription factors were identified from hard (S4) to mature (S6) fruit stage (Fig. 6b), and 21 *AcbHLHs* and seven *AcbZIPs* transcription factors were identified from young (S2) to mature (S6) fruit (Fig. 6c). During the entire fruit development period, the two transcription factor families had both positive and negative regulation on sugar structure genes.

The transcriptional expression of genes involved in acid metabolism pathway is regulated by MYB, C2H2, and GRAS transcription factors. In the present experiment, 23 MYBs, 9 *C2H2s* and 1 GRAS transcription factors were annotated during the fruit develop tment of *A. carambola*. From young (S2) to hard (S4) fruit stage, a total of 18 *AcMYBs* transcription factors were annotated, of which *AcMYB1* was separated from the other transcription factors (Fig. 6d). From hard (S4) to mature (S6) fruit stage, a total of 12 *AcMYBs*, four *AcC2H2s*, and one *AcGRAS* transcription factors were annotated, with 3 transcription factor families negatively regulated organic acid structure genes (Fig. 6e). From young (S2) to mature (S6) fruit, a total of six *AcC2H2s* and one *AcGRAS* transcription factors were annotated, and 14 organic acid structure genes were regulated both positively and negatively (Fig. 6f).

### Identification of differentially expressed genes by qRT-PCR

**Fig. 7:**
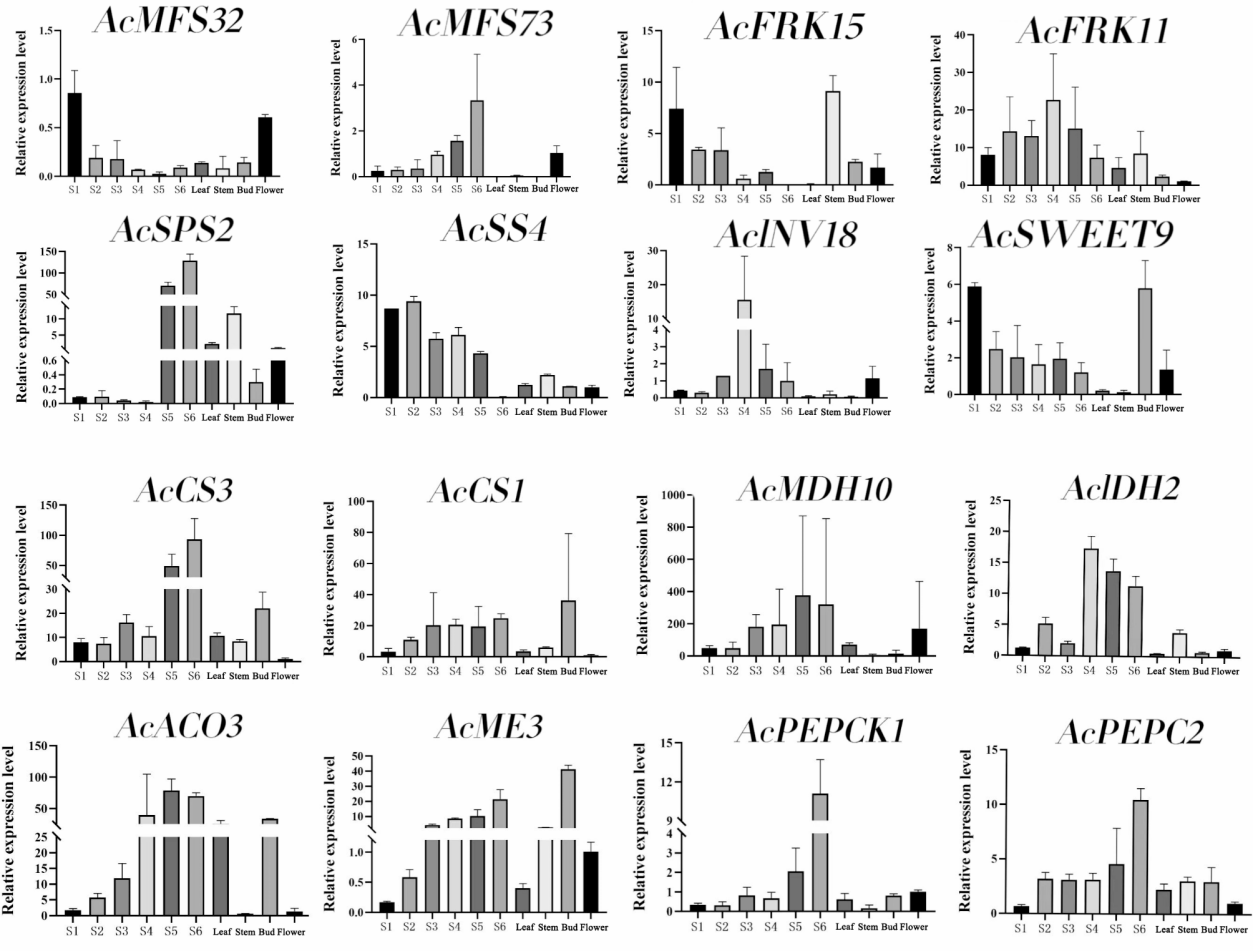
Expression patterns of genes related to sugar and organic acid metabolism and biosynthesis in different stages and tissue

In order to verify the relative expression pattern of unigenes, according to the differential expression results of metabolic group and transcriptional group, eight DEGs of glucose metabolism (*AcMFS32*,* AcMFS73*,* AcFRK11*,* AcFRK15*,* AcSPS2*,* AcSS4*,* AcINV18,* and *AcSWEET9*) and eight DEGs of organic acid metabolism (*AcCS1*,* AcCS3*,* AcMDH10*,* AcIDH2*,* AcACO3*,* AcME3*,* AcPEPCK1*, and *AcPEPC2*) were selected for qRT-PCR verification. The results showed that most of the genes were specifically expressed in tissues, such as *AcFRK15* was the highest in stem, *AcPEPCK1* in fruit mature stage (S6), *AcSPS2* in fruit mature stage (S6), leaf and stem was significantly higher than that in young fruit stage (S2), *AcINV18* in hard fruit stage (S4), and *AcCS3*, *AcACO3*, *AcME3* and *AcPEPCK1* in fruit mature stage (S6) (Fig. 7). The expression trend was consistent with the expression pattern of transcriptome data, thus verifying the reliability of transcriptome data.

## Discussion

The sugar and organic acid contents produced during fruit development and maturity can improve its quality and flavor. Therefore, it is essential to clarify the metabolic pathways and regulatory factors that govern the synthesis and accumulation of key flavor compounds in star fruit. It is very important to provide a new theoretical basis for improving the flavor quality of *A. carambola*. Based on the *A. carambola* genome, this study combined metabolic grouping and transcriptome data to screen the candidate genes related to sugar and organic acid metabolism in star fruit. This approach opened new avenues to develop the flavor and quality of star fruit.

### Potential factors affecting the changes of sugar and organic acid content in star fruits in different periods

Sugar is an important factor determining fruit quality, and the content of soluble sugar in fruit is an important evaluation index of fruit senescence and quality deterioration [40]. In this study, it was found that sucrose, fructose, and glucose were the main sugars in star fruit. The soluble sugar increased slowly during the entire fruit development process. Glucose and fructose contents were the highest in the young stage (S2), while sucrose content increased in the mature stage. Fructose content was the highest throughout the entire fruit development process. It is speculated that star fruit is a type of hexose accumulation fruits, meaning that the sugar content of star fruit varies in different periods.

The total amount and composition of organic acids are the main factors affecting fruit quality, which can directly affect its taste, flavor and sensory quality. Organic acids also play a role in accelerating digestion and maintaining the acid-base balance in the body, thereby providing health benefits [41]. Previous studies have shown that oxalic and malic acids are the main organic acids in *A. carambola*. Oxalic acid, being a toxin and anti-nutritional factor, can directly or indirectly harm human health [42]. It is worth noting that the titratable acid content in star fruit decreased rapidly with fruit ripening, with the highest contents being quinic, citric, shikimic, and malic acids. Oxalic and malic acids constitute the primary organic acids in star fruit with oxalic acid content being the highest, while citric, succinic, and maleic acids are undetectable [43]. Interestingly, oxalic acid was not found in the present study. This absence may be due to its low proportion throughout in the entire fruit ripening process. Alternatively, it could be attributed to the change of the oxalic acid’s carboxyl group methylation, leading to the formation of other substances. This discrepancy may also be related to varietal characteristics.

The expression analysis of sugar and acid metabolites revealed that most sugar metabolites were highly expressed during fruit ripening, including sucrose, maltose, melezitose, among others. Conversely, most organic acid metabolites were expressed in the early stage of fruit development. However, the accumulation rate during fruit ripening decreased significantly for metabolites such as quinic acid, maltose, citric acid, resulting in a decrease of fruit organic acid content. This phenomenon may be affected by the expression of genes related to organic acid metabolism and the activity of related enzymes [44]. Throughout the entire fruit growth process, organic acids play roles in photosynthesis, respiration, and the synthesis of sugars, phenols, lipids, aromatics, and other cellular metabolic processes. Particularly in the mature stage of fruit growth, there is a decrease in organic acid contents. Organic acids can combine with metal cations such as K⁺, Na⁺, Mg²⁺, and Ca²⁺ in fruit cells to form strong alkali and weak acid salts, thereby reducing acidity. Additionally, sugar and acid metabolites are influenced by transcriptional regulatory factors that regulate organic acid metabolism. Moreover, they may be affected by the growth environment and cultivation management measures. Collectively, these factors potentially influence the process of organic acid metabolism in fruits.

### Effect of sugar and acid expression genes on *A. carambola* fruit flavor

The accumulation of soluble sugar in fruit is a complex biological process regulated by many gene families, including invertase (INV), sucrose synthase (SS), fructokinase (FK), hexokinase (HK), sucrose phosphate synthase (SPS), and sugar transporters. Sucrose phosphate synthase (SPS) catalyzes sucrose synthesis, and its activity has been found to be positively correlated with sucrose accumulation in tomato [45]. In this study, it was found that *AcSPS2* and *AcSPS4* exhibited relatively high expression levels in the mature stage, which were consistent with the trend of sucrose accumulation. We speculate that they are key genes involved in sucrose accumulation, a conclusion consistent with previous studies. Sucrose synthase (SS) can catalyze both sucrose synthesis and sucrose decomposition. Positive correlation between SS activity and sucrose content have been reported in cherry tomato fruit, while negative correlation between SS activity and sucrose content have been reported in jujube (*Ziziphus jujuba*) was reported [46, 47]. Additionally, the expression levels of *AcSS2* and *AcSS4* decreased during fruit ripening, suggesting a role in sucrose decomposition. Conversely, e *AcSS3* expression increased during fruit ripening, presumed to be the key enzyme involved in sucrose accumulation. Furthermore, the expression level of most *AcINVs* was high in the young and hard fruit stage, and decreasing significantly in the mature stage, results consistent with the high activity of INV in the fast-growing young tissues and meristems. The results of sugar content determination from the metabolic group showed that fructose and glucose contents were the highest in the young fruit stage. This finding is consistent with the conclusion that invertase (INV) enzyme activity increased, leading to the accumulation of fructose and glucose mainly in pineapple from the young to rapid fruit growth period [48]. Hexokinase (HXK) plays an important role in regulating cell carbon flow and energy state. In this study, the expression of *AcHXK2* was higher in the young fruit stage and decreased gradually with fruit development. This pattern may be related to the early use of sugar to maintain the metabolic activity of the fruit itself. However, with fruit ripening, sugar utilization decreased, gradually transitioning to the process of accumulation. A similar conclusion was reported in apple fruit, where the expression level of hexokinase decreased with the decrease in energy utilization or tricarboxylic acid cycle activity [49]. Additionally, *AcFRK8* and *AcFRK15* were highly expressed in the early stage of fruit development, suggesting they are important candidate genes for fructose accumulation.

Phosphoenolpyruvate carboxylase (PEPC) and citrate synthase (CS) are key enzymes in citric acid synthesis. For example, the activities of CS and PEPC were positively correlated with fruit organic acid content in citrus cultivars [50]. However, other studies showed that there is no correlation between CS activity and citrate accumulation in fruits [51]. In this study, *AcPEPC1* and three *AcCSs* genes were highly expressed at the ripening stage (S6), while the content of citric acid decreased with fruit development and ripening, suggesting that the downstream enzymes of lemon metabolism could determine the accumulation and decomposition of citric acid. Additionally, aconitase (ACO) can degrade citric acid in the cytoplasm, and an increase in cis-aconitase activity leads to decrease of citric acid [52]. Furthermore, it was found that *AcACO1* and *AcACO3* were highly expressed in the mature stage, indicating their potential involvement in citric acid degradation. Malic enzyme (ME) is the key enzyme in the glyoxylic acid cycle and plays an important role in different processes of plant growth and development. In grape (*Vitis vinifera*), ME was associated with the degradation of malic acid during fruit ripening [29]. Here, *AcME3* was highly expressed during fruit ripening. The results of organic acid determination in metabolic group showed that the overall content of malic acid decreased during fruit development, suggesting a potential correlation between *AcME3* expression and malic acid degradation. Cytoplasmic NAD-dependent malate dehydrogenase (NAD-MDH) is mainly involved in malate synthesis, and malate accumulation in kiwifruit mainly depends on the increase in the activity of NAD-MDH. *AeCS2* and *AeMDH2* play a key role in controlling the activity of citrate synthase (CS) and NAD-MDH, respectively [53]. It is worth noting that in star fruit, the expression of *AcMDH7* is high in the early stage of fruit development, suggesting a potential role in regulating malic acid accumulation. *AcMDH9* and *AcMDH10* have the highest expression during fruit ripening, indicating they may not participate significantly in malic acid accumulation or have minimal regulation on malic acid levels. The highly expressed *AcMDH4* gene in the hard fruit stage suggests its importance in the tricarboxylic acid (TCA) cycle and basic energy metabolism. Aluminum-activated malate transporter (ALMT) is a plant-specific anion channel protein. that transports malic acid into vacuoles in the form of malate anions [54]. The expression levels of *AcALMT5*, *AcALMT6*, *AcALMT7* and *AcALMT11* in star fruit decreased gradually with fruit ripening, which is consistent with the observed decrease of malic acid content in the middle and later stages of star fruit development. Other *AcALMTs* were almost not expressed in the fruit. Yang et al. [55] found that the expression levels of VHA-An and *VHP1* in loquat fruits treated with low acid were significantly higher than those treated with high acid, consistent with the leakage hypothesis proposed by Echeverria et al. [56]. This hypothesis suggests that star fruit may have high vacuole leakage at low acidity, leading to an increase in the expression of tonoplast proton pumps to compensate for the proton leakage.

### Regulatory role of transcription factors in sugar and organic acid metabolism of star fruit

Sucrose phosphate synthases (SPS) is the main enzyme in sucrose synthesis and plays an important role in regulating sucrose synthesis and its accumulation. It has been identified in several plants with varying members of numbers, such as *Arabidopsis thaliana* (6), *Populus trichocarpa* (7), corn (3), potato (4), rice (7) [57]. Four SPS genes were identified in star fruit, showing high homology with *Arabidopsis thaliana* and apple, indicating relative conservation in their evolutionary process. Sucrose synthase (SS) is the key enzyme regulating sucrose metabolism, affecting biomass formation and sugar accumulation throughout the plant growth and development process [58, 62]. The number of SS genes varies among plants, for example, maize (5), *Arabidopsis thaliana* (6), and rice and millet (9).In this study, seven SS genes were screened, and the phylogenetic tree was constructed with *Arabidopsis thaliana* and cassava. It was found that the sucrose synthase gene of star fruit had high homology with other sequences, indicating that the sequence is also conserved. Ten and six hexokinases (HXK) members were reported in rice and Arabidopsis, respectively [58, 59], while star fruit has three genes obtained by bioinformatics analysis. The number of HXK genes varied greatly among species, indicating that the gene was not evolutionary conserved. According to previous studies, the number of fructokinases (FRK) genes in *Monocotyledon sorghum*, rice, and corn is 6, 8 and 8, respectively, while the FRK members of dicotyledonous plant varied (*Arabidopsis* (12), grape (9), and tomato (8)) [60–62]. In this study, 15 FRK genes were identified from the *A. carambola* genome. It is observable that the number of FRK members in monocotyledons is typically fewer than in dicotyledons. This suggests that the evolution of FRK genes in dicotyledons occurs at a faster pace, with a more pronounced differentiation of homologous genes. Additionally, only four sucrose transporter genes were identified in *A. carambola* genome. It is speculated that the *A. carambola* genome has a small sucrose transporter family. In the phylogenetic analysis of the SUC and SWEET families, some branches of the evolutionary tree contain only *Arabidopsis thaliana* gene family, which may be associated with the active sugar transporter family. The gene structure of *AtSUCs* and *AcSWEETs* further confirms this view. The number of exons of the four *AtSUCs* genes varies considerably. Additionally, their arrangement differs significantly, indicating varying degrees of exon and intron increase or loss, insertion or deletion events during evolution.

A total of 72 acid metabolism-related genes were identified in the genome of star fruit. The results revealed that most acid metabolism-related gene families exist in small families. While the identification of gene families has been reported in citrus [63] and pear [64], research on acid metabolism lags behind that of sugar metabolism. The citrate synthase and malate synthase gene families of *A. carambola*, *Arabidopsis thaliana*, citrus, and papaya were constructed to build an evolutionary tree. The results revealed that the citrate synthase family in A. carambola is similar to that of other species with high homology, whereas the members of the malate synthase family are distinct from those of other species, exhibiting reduced similarity. This suggests that the gene may have undergone rapid evolution.

### Gene network regulates the pathway of sugar and acid metabolism in star fruit

Pairwise comparison of all differentially expressed genes (DEGs) and differentially accumulated metabolites (DAMs) in the three periods (S2, S4, S6) indicated that the KEGG pathway was mainly enriched in metabolic pathways and biosynthesis pathways of secondary metabolites. In the sugar metabolism pathway, the DEGs related to sugar synthesis are mainly enriched in starch and sucrose metabolism, fructose and mannose metabolism, amino sugar and nucleotide sugar metabolism, and galactose metabolism. The DAMs are enriched in starch and sucrose metabolism and the phosphoglucosyl transferase system (PTS) metabolism pathway. PTS, as a sugar transport system, not only mediates carbohydrate uptake and phosphorylation but also participates in the regulation of central carbon and nitrogen metabolism. Several studies have shown a close relationship between carbohydrate and acid metabolism [65]. In the pathway of acid metabolism, the DEGs related to organic acids are significantly enriched in carbon metabolism, carbon fixation of photosynthetic organisms, microbial metabolism in different environments, and the tricarboxylic acid cycle. DAMs are mainly enriched in metabolic pathways and biosynthesis of secondary metabolites, followed by phenylpropanoid biosynthesis, carbon metabolism, and the tricarboxylic acid cycle, which are closely related to sugar and organic acid metabolism.

The seven core candidate genes in glucose metabolism pathway include *AcFRK8*,* AcFRK15*,* AcINV3*,* AcINV14*,* AcSS2*,* AcSS4*, and *AcSPS4*. INV, SS, and SPS are common structural genes in sucrose metabolism. It has been reported that *AtSUS2* is mainly involved in *Arabidopsis thaliana* seed maturation [66]. In this study, *AcSS2* has high homology with *AtSS2*. *AcSS2* is expressed in the young and almost did not express in the mature stage. It is speculated that *AcSS2* has similar function with *AtSS2*. Studies have shown that SPS gene family is highly expressed in most plants’ fruits, and the expression abundance increased with fruit growth and development (e.g., pineapple, peach, pear, apple and citrus) [67, 68]. *AcSPS4* is highly expressed during fruit ripening, indicating that *AcSPS4* plays an important role in sucrose accumulation in star fruit. FRK is mainly involved in plant fructose metabolism, in this study, *AcFRK8* and *AcFRK15* were highly expressed in young fruit, suggesting that it could promote fructose accumulation in the early stage of fruit development. The 17 core candidate genes in acid metabolism pathway (*AcACO1*,* AcACO3*,* AcALMT7*,* AcALMT11*,* AcME3*,* AcGAD1*,* AcGAD2*,* AcMDH4*,* AcMDH9*,* AcMDH10*,* AcVHP1*,* AcPIDH1*,* AcCS1*, *AcCS2*,* AcCS4*,* AcPEPC1*, and *AcGADPH5*), including 10 structural genes are involved in tricarboxylic acid metabolism. It is suggested that these genes are key genes involved in citric, malic and succinic acids metabolism.In the future, it will be essential to focus on cloning these highly expressed sugar and acid metabolism genes for functional validation.

### Regulatory role of transcription factors in sugar and organic acid metabolism of star fruit

At present, 78 and 157 bZIP transcription factor genes were identified in plum and peach, respectively [69, 70]. In contrast, only 8 were predicted in star fruit, indicating that these transcription factors were involved in the fruit important biological functions regulation. Because bZIP participates in many metabolic processes such as stress, signal transduction, and plant growth [71], it is suggested that some bZIP transcription factors may not be annotated and may also be expressed in other tissues and organs of *A. carambola*. AtbZIP63 in *Arabidopsis thaliana* has an important node in the interaction network between glucose and ABA [72], which is regulated by both ABA and glucose. *AtbZIP63* and its homologue *AtbZIP3* can synergistically inhibit the interaction between glucose and ABA, thus inhibiting glucose content and regulating the stability of soluble sugar content in fruit. In this study, it was found that most *AcbZIPs* genes were positively correlated with sucrose synthase and fructokinase expression, indicating that they played a positive role in the accumulation of fructose regulation. In addition, we found that a class of transcription factor family bHLH, which is opposite to the expression of bZIP transcription factor family, has 29 *AcbHLH* genes annotated during the entire star fruit development. This transcription factor is positively correlated with the expression of invertase and sucrose phosphate synthase, indicating that bHLH is positively correlated with sucrose accumulation. It is suggested that bZIP and bHLH transcription factor families can cooperatively inhibit the transformation between sucrose and fructose, thus regulating the balance of sugar content in the fruit.

Transcription factor MYB is closely related to fruit quality. Malic acid content of transcription factor *MdMYB44* is significantly correlated in apple [73]. Overexpression of *CrMYB73* in citrus is beneficial to citric acid accumulation in fruit [74]. In this study, a total of 23 MYB transcription factors were annotated, which regulate both positive and reverse genes of organic acid structure. Other transcription factors are also involved in the biosynthesis of organic acids. C2H2 zinc finger structural proteins are widely involved in plant organ morphogenesis, hormone signal transduction, stress response, among other functions [75]. Nine C2H2 transcription factors were annotated in star fruit. GRAS is a unique family of transcription factors in plants, which plays an important role in plant response to stress and growth and development [76]. Only one GRAS gene was predicted in star fruit. It is speculated that this transcription factor may not be annotated, or it may have little effect on acid metabolism.

## Conclusion

In this study, we integrated transcriptome and metabolome data to elucidate the metabolic pathways of sugar and organic acid utilization in *A. carambola* using the species genome. We identified eight candidate genes for sugar metabolism and eight for organic acid metabolism. Additionally, we predicted that two transcription factors had effects on sugar metabolism, while 14 transcription factors were associated with organic acid metabolism. Furthermore, we analyzed the gene evolution in *A. carambola* sugar and organic acid biosynthesis pathways, providing, for the first time, a new understanding of the molecular mechanism underlying *A. carambola* sugar and organic acid biosynthesis. This study offers a powerful foundation for improving *A. carambola* sugar and organic acid biosynthesis pathways and verifying related genes in future research endeavors.

## Materials and methods

### Plant material collection

The *A. carambola* ‘Beiliusuan No. 1’ variety was selected from the germplasm collection of the Institute of Horticulture, Guangxi Academy of Agricultural Sciences, Nanning City, Guangxi Province, China, as research material. Three star fruit trees with good and similar growth and free from diseases and insects were sampled. According to the external color and size uniformity of each fruit, samples were collected from the green fruit (S1, 10 days after anthesis), young fruit (S2, 20 days after anthesis), expansion (S3, 30 days after anthesis), hard fruit (S4, 40 days after anthesis), color conversion (S5, 50 days after anthesis), and maturity stages (S6, 60 days after anthesis). Samples were collected every 10 days during the morning period. Fruits surface was rinsed with 70% alcohol and RNA inhibitor before harvest, and were cut into small pieces after harvest (small fruit 1 cm x 1 cm; hard fruit 2 cm × 2 cm; mature fruit 4 cm × 4 cm) and were quickly frozen in liquid nitrogen and stored at -80 ℃ until further use.

### Physiological indices measurement

In the same period, a total of 20-star fruits were randomly selected and each single fruit weight was determined, fruit vertical and transverse diameters were measured with Vernier caliper, and fruit shape index (vertical diameter/horizontal diameter) was calculated. Several physiological indices were determined as follows: titratable acid content (Titratable acidity, TA) measured by NaOH neutralization titratable method, a burette was used to slowly add NaOH solution to the sample, which had been treated with phenolphthalein indicator, while continuously stirring. The titration was continued until the sample changed color from colorless to deep red. and total soluble sugar content (Soluble sugar, SS) measured by the anthrone concentrated sulfuric acid method with each measurement repeated three times for each fruit. The sugar to acid ratio (RST: SS/TA) was calculated, and fruit hardness was measured by hardness tester [77, 78]. Data were analyzed by Excel and SPSS17.0 software.

### RNA extraction and sequencing

According to the characteristics of polysaccharides and polyphenols in star fruit, the polysaccharide polyphenol kit was used to extract the fruit tissue, and the extraction process was repeated three times for each period [79]. The extracted RNA samples were initially detected by 1% agarose gel electrophoresis, and then the integrity of RNA was further tested, including RNA purity (OD_260_/OD_280_), RNA concentration, RIN value, 28 S/18S, map baseline. After the RNA samples were extracted, cDNA library was established, and total RNA was transformed into fragment sequence by purification and splicing, and sequenced by Illumina high-throughput sequencer. The quality of the constructed library was tested, and chain-specific library was constructed with the extracted RNA. The HiSeq2500 ultra-high throughput sequencer was used for double-terminal sequencing, with reading length of 100nt. After sequencing, the original transcriptome data were analyzed and processed, followed by sequence assembly, unigenes structure annotation, and functional classification.

### Metabolome sampling extraction, qualitative and quantitative analyses

Firstly, the selected star fruits were freeze-dried in vacuum, and the treated samples were ground to powder by a grinder (30 Hz for 1.5 min). The sample powder of 100 mg was dissolved in 1.0mL extract and the dissolved sample was refrigerated overnight at 4 ℃. During this period, the sample was spun three times to improve the extraction rate. After overnight sample centrifugation (rotating speed 10,000 g, 10 min), the supernatant was absorbed, the sample was filtered by microporous membrane and stored in the injection bottle for or LC-MS/MS analysis. The software Analyst1.6.3 was used to process the mass spectrometry data. Based on the local metabolic database and mass spectrometry data, samples’ metabolites were qualitatively and quantitatively analyzed.

### Functional annotation and classification of unigenes

Unigenes’ sequences were compared by blast to the protein database Nr, Swiss-Prot, KEGG, and COG of NCBI (National Center for Biotechnology Information) (E-value < 0.0001). Concurrently, unigenes sequences were compared by blastn in the nucleic acid database NT on NCBI, and the protein with the highest similarity to unigenes’ sequences were obtained to fully understand their functional annotation information.

### Unigenes functional annotation and classification

The mapping results were quantified by cufflinks (version:2.1.1). According to the expression value FPKM (Fragments Per Kilobase of transcript per Million) of star fruit gene in each transcriptome sample calculated by cufflinks, the correlation map reflecting the biological repetition of each sample was drawn by R language, PCA (Principal Component Analysis) was carried out with fast.prcomp function in R language gmodels package, and scatter plot showing the correlation of biological repetition among samples was drawn. Samples from three different periods were compared and screened for differentially expressed genes (DEGs). Fold-change (multiple of expression difference) and Fisher’s exact test statistics were used to screen the differences among DEGs, and the screening condition position FDR (error detection rate) < 0.01 and Fold-change > 2. Then the differential genes with the same expression trend in the three groups were collected, and up- or down-regulated differential genes in different fruit stages in the three groups were extracted. For the KEGG enrichment analysis of DEGs, cDNA or protein sequences of all the identified DEGs were compared to the KEGG database (http://www.genome.jp/kegg/)) using the Blast software in Kobas to obtain the KO number and then extracted the KEGG pathway to which the gene belongs. The corrected *P* < 0.05 was selected as the threshold to determine the statistically significant enrichment in the gene set.

### Alignment of reference genomes and phylogenetic analysis of genes related to sugar and acid metabolism

The family of genes related to sugar and acid metabolism in star fruit was obtained from *A. carambola* whole genome sequence [80]. Combined with the literature and related genes regulating sugar and acid metabolism, similar genes in other species were classified and sorted. The genome data of star fruit were compared to other species by BLASTP software. HMM model of related gene families was download from Pfam (http://pfam.xfam.org/) database, and then searched in HMM3.0 software to search the local database with e value less than 10 − 5, and screen the related gene ID and protein sequences containing these domains in *A. carambola*. Then, the protein sequences obtained by the two methods were integrated, the redundant terms were removed and incomplete sequencing were deleted. The integrity of conserved domains was checked by SMART (http://smart.embl.de/) and NCBI-CDD (https://www.ncbi.nlm.nih.gov/cdd) databases, and genes that did not contain related domains and did not accord with the characteristics of related gene families were removed. Full-length amino acid sequences of the related proteins of other species were obtained from the NCBI database (http://www.ncbi.nlm.nih.gov/protein/), and then multiple sequence alignment (using ClustalW program) [81] was carried out by MEGAv7.0.26 software. The phylogenetic tree was constructed by the adjacent trees method. The bootstrap method value was repeated 1,000 times, and other parameters were set to default values.

#### Prediction of transcription factors of candidate genes and construction of protein interaction network

The protein sequences of sugar and acid candidate genes were uploaded to plantTFDB plant transcription factor database (http://planttfdb.cbi.Pku.edu.cn/prediction.php) to predict the existence of transcription factors. The protein interaction network of structural genes and transcription factors in *A. carambola* was constructed by STRING website (http://string-db.org/), and the possible protein interaction form of co-expression genes in *A. carambola* was inferred.

### Quantitative RT*-*PCR verification of differentially expressed genes

After confirming that the extracted total RNA was qualified, it was reverse transcribed to synthesize cDNA using a reverse transcription kit, and the obtained cDNA was stored at -20 ℃. Combined with the results of genome and transcriptome screening, the primers of eight sugar and eight acid related genes were designed using Primer5.0 software according to the internal reference gene ACT- β gene [82] (Supplementary Table 1). The relative expression level was calculated by 2^−∆∆CT^ method, and the gene expression level was calculated by Prism software. Primer parameter setting: TM value is 58 ℃, primer length is 19 ℃, primer length is 22 BP, GC content is 45-55%, and dimer and hairpin structure are avoided.

## Electronic supplementary material

Below is the link to the electronic supplementary material.

Supplementary Material 1

Supplementary Material 2

Supplementary Material 3

Supplementary Material 4

Supplementary Material 5

Supplementary Material 6

Supplementary Material 7

## Data Availability

Sequence data that support the findings of this study have been deposited in the National Center for Biotechnology Information with the primary accession code PRJWB13140.

## References

[1] Fieischmann P, Watanabe N, Winterhalter P. Enzymatic carotenoid cleavage in star fruit (*Averrhoa carambola*). Phytochemistry. 2003;63:131–7. 12711133 10.1016/s0031-9422(02)00657-x

[2] Luan F, Peng L, Lei Z, et al. Traditional uses, Phytochemical constituents and Pharmacological properties of *Averrhoa carambola* L.: a review. Front Pharmacol. 2021;12:699–899. 10.3389/fphar.2021.699899 PMC8407000 34475822

[3] Giovannoni JJ. Genetic regulation of fruit development and ripening. Plant Cell. 2004;16:170–80. 10.1105/tpc.019158 PMC2643394 15010516

[4] Durán-Soria S, Pott DM, Osorio S, et al. Sugar signaling during fruit ripening. Front Plant Sci. 2020;11:564–917. 32983216 10.3389/fpls.2020.564917 PMC7485278

[5] Koch K. Sucrose metabolism: regulatory mechanisms and pivotal roles in sugar sensing and plant development. Curr Opin Plant Biol. 2004;7:235–46. 15134743 10.1016/j.pbi.2004.03.014

[6] Chen T, Zhang Z, Li B, et al. Molecular basis for optimizing sugar metabolism and transport during fruit development. aBIOTECH. 2021;2:330–40. 36303881 10.1007/s42994-021-00061-2 PMC9590571

[7] Liu Z, Franks RG. Molecular basis of fruit development. Front Plant Sci. 2015;6:28. 25699063 10.3389/fpls.2015.00028 PMC4318284

[8] Ilyas RA, Sapuan SM, Ishak MR, et al. Development and characterization of sugar palm nanocrystalline cellulose reinforced sugar palm starch bionanocomposites. Carbohydr Polym. 2018;202:186–202. 30286991 10.1016/j.carbpol.2018.09.002

[9] Ruan YL. Sucrose metabolism: gateway to diverse carbon use and sugar signaling. Annu Rev Plant Biol. 2014;65:33–67. 24579990 10.1146/annurev-arplant-050213-040251

[10] Ruan YL, Jin Y, Yang YJ, et al. Sugar input, metabolism, and signaling mediated by invertase: roles in development, yield potential, and response to drought and heat. Mol Plant. 2010;3:942–55. 20729475 10.1093/mp/ssq044

[11] Ma P, Zhang X, Chen L, et al. Comparative analysis of sucrose phosphate synthase (SPS) gene family between *Saccharum officinarum* and *Saccharum spontaneum*. BMC Plant Biol. 2020;20:422. 32928111 10.1186/s12870-020-02599-7 PMC7488781

[12] Li C, Zhang M, Qi N, et al. Abscisic acid induces adventitious rooting in cucumber (*Cucumis sativus* L.) by enhancing sugar synthesis. Plants (Basel). 2022;11:2354. 36145755 10.3390/plants11182354 PMC9505232

[13] Cheng J, Wen S, Xiao S, et al. Overexpression of the tonoplast sugar transporter CmTST2 in melon fruit increases sugar accumulation. J Exp Bot. 2018;69:511–23. 29309616 10.1093/jxb/erx440 PMC5853577

[14] Wen S, Neuhaus HE, Cheng J, et al. Contributions of sugar transporters to crop yield and fruit quality. J Exp Bot. 2022;73:2275–89. 35139196 10.1093/jxb/erac043

[15] Büttner M. The Arabidopsis sugar transporter (AtSTP) family: an update. Plant Biol (Stuttg). 2010;12:35–41. 10.1111/j.1438-8677.2010.00383.x 20712619

[16] Chaudhuri B, Hörmann F, Lalonde S, et al. Protonophore- and pH-insensitive glucose and sucrose accumulation detected by FRET nanosensors in Arabidopsis root tips. Plant J. 2008;56:948–62. 18702670 10.1111/j.1365-313X.2008.03652.x PMC2752219

[17] Afoufa-Bastien D, Medici A, Jeauffre J, et al. The *Vitis vinifera* sugar transporter gene family: phylogenetic overview and macroarray expression profiling. BMC Plant Biol. 2010;10:245. 21073695 10.1186/1471-2229-10-245 PMC3095327

[18] Hackel A, Schauer N, Carrari F, et al. Sucrose transporter LeSUT1 and LeSUT2 inhibition affects tomato fruit development in different ways. Plant J. 2006;45:180–92. 16367963 10.1111/j.1365-313X.2005.02572.x

[19] Cai Y, Yan J, Tu W, et al. Expression of sucrose transporters from *Vitis vinifera* Confer High Yield and enhances Drought Resistance in Arabidopsis. Int J Mol Sci. 2020;21:2624. 32283825 10.3390/ijms21072624 PMC7177370

[20] Sun L, Deng R, Liu J, et al. An overview of sucrose transporter (SUT) genes family in rice. Mol Biol Rep. 2022;49:5685–95. 35699859 10.1007/s11033-022-07611-x

[21] Patil G, Valliyodan B, Deshmukh R, et al. Soybean (*Glycine max*) SWEET gene family: insights through comparative genomics, transcriptome profiling and whole genome re-sequence analysis. BMC Genomics. 2015;16:520. 26162601 10.1186/s12864-015-1730-y PMC4499210

[22] Li W, Ren Z, Wang Z, et al. Evolution and stress responses of *Gossypium hirsutum* SWEET genes. Int J Mol Sci. 2018;19:769. 29517986 10.3390/ijms19030769 PMC5877630

[23] Jian H, Lu K, Yang B, et al. Genome-wide analysis and expression profiling of the SUC and SWEET gene families of sucrose transporters in oilseed rape (*Brassica napus* L). Front Plant Sci. 2016;7:1464. 27733861 10.3389/fpls.2016.01464 PMC5039336

[24] Lee CP, Elsässer M, Fuchs P, et al. The versatility of plant organic acid metabolism in leaves is underpinned by mitochondrial malate-citrate exchange. Plant Cell. 2021;33:3700–20. 34498076 10.1093/plcell/koab223 PMC8643697

[25] Moormann J, Heinemann B, Hildebrandt TM. News about amino acid metabolism in plant-microbe interactions. Trends Biochem Sci. 2022;47:839–50. 35927139 10.1016/j.tibs.2022.07.001

[26] Zhao J, Shen F, Gao Y, et al. Parallel bud mutation sequencing reveals that fruit sugar and acid metabolism potentially influence stress in malus. Int J Mol Sci. 2019;20:5988. 31795097 10.3390/ijms20235988 PMC6928686

[27] Jiang CC, Fang ZZ, Zhou DR, et al. Changes in secondary metabolites, organic acids and soluble sugars during the development of plum fruit cv. ‘Furongli’ (*Prunus salicina Lindl*). J Sci Food Agric. 2019;99:1010–9. 30009532 10.1002/jsfa.9265

[28] López-Millán AF, Morales F, Abadía A, et al. Changes induced by Fe deficiency and Fe resupply in the organic acid metabolism of sugar beet (*Beta vulgaris*) leaves. Physiol Plant. 2001;112:31–8. 11319012 10.1034/j.1399-3054.2001.1120105.x

[29] Sweetman C, Deluc LG, Cramer GR, et al. Regulation of malate metabolism in grape berry and other developing fruits. Phytochemistry. 2009;70:1329–44. 19762054 10.1016/j.phytochem.2009.08.006

[30] Han S, Liu H, Han Y, et al. Effects of calcium treatment on malate metabolism and γ-aminobutyric acid (GABA) pathway in postharvest apple fruit. Food Chem. 2021;334:127479. 32688181 10.1016/j.foodchem.2020.127479

[31] Lama K, Peer R, Shlizerman L, et al. Tissue-specific organic acid metabolism in reproductive and non-reproductive parts of the fig fruit is partially induced by pollination. Physiol Plant. 2020;168:133–47. 30740711 10.1111/ppl.12941

[32] Chen W, Gong L, Guo Z, et al. A novel integrated method for large-scale detection, identification, and quantification of widely targeted metabolites: application in the study of rice metabolomics. Mol Plant. 2013;6:1769–80. 23702596 10.1093/mp/sst080

[33] Cho K, Cho KS, Sohn HB, et al. Network analysis of the metabolome and transcriptome reveals novel regulation of potato pigmentation. J Exp Bot. 2016;67:1519–33. 26733692 10.1093/jxb/erv549 PMC4762390

[34] Nagashima Y, He K, Singh J, et al. Transition of aromatic volatile and transcriptome profiles during melon fruit ripening. Plant Sci. 2021;304:110809. 33568307 10.1016/j.plantsci.2020.110809

[35] Wang RC, Shu P, Zhang C, et al. Integrative analyses of metabolome and genome-wide transcriptome reveal the regulatory network governing flavor formation in kiwifruit (*Actinidia chinensis*). New Phytol. 2021;33:373–89. 10.1111/nph.17618 34255862

[36] Wang Z, Song M, Wang Z, et al. Metabolome and transcriptome analysis of flavor components and flavonoid biosynthesis in fig female flower tissues (*Ficus carica* L.) after bagging. BMC Plant Biol. 2021;21:1–14. 34433422 10.1186/s12870-021-03169-1 PMC8386004

[37] Umer MJ, Safdar LB, Gebremeskel H et al. Identification of key gene networks controlling organic acid and sugar metabolism during watermelon fruit development by integrating metabolic phenotypes and gene expression profiles. Hortic Res. 2020;7. 10.1038/s41438-020-00416-8 PMC7705761 33328462

[38] Wu X, Shi X, Bai M, et al. Transcriptomic and gas chromatography-mass spectrometry metabolomic profiling analysis of epidermis provides insights into cuticular wax regulation in developing ‘Yuluxiang’ pear fruit. J Agric Food Chem. 2019;67:8319–31. 31287308 10.1021/acs.jafc.9b01899

[39] Wang RC, Shu P, Zhang C, et al. Integrative analyses of metabolome and genome-wide transcriptome reveal the regulatory network governing flavor formation in kiwifruit (*Actinidia chinensis*). New Phytol. 2022;233:373–89. 34255862 10.1111/nph.17618

[40] Edoardo Vignati M, Lipska JM, Dunwell, et al. Fruit Dev Sweet Cherry Plants. 2022;11:1531–1531. 10.3390/plants11121531 PMC9227597 35736682

[41] Walker RP, Famiani F. Organic acids in fruits: metabolism, functions and contents. Hortic Reviews. 2018;45:371–430.

[42] Jia X, Yang D, Yang Y, et al. Carotenoid-derived flavor precursors from *Averrhoa carambola* fresh fruit. Molecules. 2019;24:256. 30641936 10.3390/molecules24020256 PMC6359364

[43] Yasawardene P, Jayarajah U, De Zoysa I, et al. Mechanisms of star fruit (*Averrhoa carambola*) toxicity: a mini-review. Toxicon. 2020;187:198–202. 32966829 10.1016/j.toxicon.2020.09.010

[44] Batista-Silva W, Nascimento VL, Medeiros DB, et al. Modifications in organic acid profiles during fruit development and ripening: correlation or causation? Front Plant Sci. 2018;9:1689. 30524461 10.3389/fpls.2018.01689 PMC6256983

[45] Kim YX, Kwon MC, Lee S, et al. Effects of nutrient and water supply during fruit development on metabolite composition in tomato fruits (*Solanum lycopersicum L.*) grown in magnesium excess soils. Front Plant Sci. 2020;11:562399. 33101331 10.3389/fpls.2020.562399 PMC7545823

[46] Sun L, Wang J, Lian L, et al. Systematic analysis of the sugar accumulation mechanism in sucrose-and hexose-accumulating cherry tomato fruits. BMC Plant Biol. 2022;22:303. 35729535 10.1186/s12870-022-03685-8 PMC9215100

[47] Wang Y, Ren S, Li X, et al. Shading inhibits sugar accumulation in leaf and fruit of jujube (*Ziziphus jujuba Mill*). Horticulturae. 2022;8:592.

[48] Lu XH, Sun DQ, Wu QS, et al. Analysis of components and contents of soluble sugars and organic acids in pineapple germplasm. J Fruit Sci. 2013;30:444–8.

[49] Li M, Feng F, Cheng L. Expression patterns of genes involved in sugar metabolism and accumulation during apple fruit development. PLoS ONE. 2012;7:e33055. 22412983 10.1371/journal.pone.0033055 PMC3296772

[50] Zhou Y, He W, Zheng W, et al. Fruit sugar and organic acid were significantly related to fruit mg of six citrus cultivars. Food Chem. 2018;259:278–85. 29680055 10.1016/j.foodchem.2018.03.102

[51] Delhaize E, Ryan PR, Hocking PJ, et al. Effects of altered citrate synthase and isocitratedehydrogenase expression on internal citrate concentrations and citrate efflux from tobacco (*Nicotiana tabacuml*) roots. Plant Soil. 2003;248:137–44.

[52] Gupta KJ, Shah JK, Brotman Y, et al. Inhibition of aconitase by nitric oxide leads to induction of the alternative oxidase and to a shift of metabolism towards biosynthesis of amino acids[J]. J Exp Bot. 2012;63:1773–84. 22371326 10.1093/jxb/ers053

[53] Jia D, Xu Z, Chen L, et al. Analysis of organic acid metabolism reveals citric acid and malic acid play major roles in determining acid quality during the development of kiwifruit (*Actinidia Eriantha*). J Sci Food Agric. 2023;103:6055–69. 37127927 10.1002/jsfa.12678

[54] Sharma T, Dreyer I, Kochian L, et al. The ALMT family of organic acid transporters in plants and their involvement in detoxification and nutrient security. Front Plant Sci. 2016;7:1488. 27757118 10.3389/fpls.2016.01488 PMC5047901

[55] Yang J, Zhang J, Niu XQ, et al. Comparative transcriptome analysis reveals key genes potentially related to organic acid and sugar accumulation in loquat. PLoS ONE. 2021;16:e0238873. 33914776 10.1371/journal.pone.0238873 PMC8084190

[56] Echeverria E, Gonzalez PC, Brune A. Characterization of proton and sugar transport at the tonoplast of sweet lime (*Citrus limmetioides*) juice cells. Physiollogia Plant. 1997;101:291–300.

[57] Xiao N, Ma H, Wang W, et al. Overexpression of ZmSUS1 increased drought resistance of maize (*Zea mays L.*) by regulating sucrose metabolism and soluble sugar content. Planta. 2024;259:1–14. 10.1007/s00425-024-04336-y 38277077

[58] Cho JI, Ryoo N, Ko S, et al. Structure, expression, and functional analysis of the hexokinase gene family in rice (*Oryza sativa* L). Planta. 2006;224:598–611. 16552590 10.1007/s00425-006-0251-y

[59] Karve A, Rauh BL, Xia X, et al. Expression and evolutionary features of the hexokinase gene family in *Arabidopsis*. Planta. 2008;228:411–25. 18481082 10.1007/s00425-008-0746-9 PMC2953952

[60] Stein O, Granot D. Plant fructokinases: evolutionary, developmental, and metabolic aspects in sink tissues. Front Plant Sci. 2018;9:339. 29616058 10.3389/fpls.2018.00339 PMC5864856

[61] Riggs JW, Cavales PC, Chapiro SM, et al. Identification and biochemical characterization of the fructokinase gene family in *Arabidopsis thaliana*. BMC Plant Biol. 2017;17:1–18. 28441933 10.1186/s12870-017-1031-5 PMC5405513

[62] Du M, Zhu Y, Nan H, et al. Regulation of sugar metabolism in fruits. Sci Hort. 2024;326:112712.

[63] Zhang M, Lu W, Li Q, et al. Comprehensive analyses of the citrus WRKY gene family involved in the metabolism of fruit sugars and organic acids. Front Plant Sci. 2023;14:1264283. 37780491 10.3389/fpls.2023.1264283 PMC10540311

[64] Li Q, Qiao X, Jia L, et al. Transcriptome and resequencing analyses provide insight into differences in organic acid accumulation in two pear varieties. Int J Mol Sci. 2021;22:9622. 34502530 10.3390/ijms22179622 PMC8456318

[65] Erni B. The bacterial phosphoenolpyruvate: sugar phosphotransferase system (PTS): an interface between energy and signal transduction. J Iran Chem Soc. 2013;10:593–630.

[66] Angeles-Núñez JG, Tiessen A. Regulation of AtSUS2 and AtSUS3 by glucose and the transcription factor LEC2 in different tissues and at different stages of Arabidopsis seed development. Plant Mol Biol. 2012;78:377–92. 22228409 10.1007/s11103-011-9871-0

[67] Johnson R. The US trade situation for fruit and vegetable products. Washington, DC: *Congressional Research Service*. 2014.

[68] Johari NHF, Dolhaji NH, Shamsuri S, et al. A review on sugar and organic profiles on the postharvest quality of fruits. Sci Lett. 2023;17:91–108.

[69] Yong X, Zheng T, Zhuo X, et al. Genome-wide identification, characterisation, and evolution of ABF/AREB subfamily in nine Rosaceae species and expression analysis in Mei (*Prunus mume*). PeerJ. 2021;9:e10785. 33604183 10.7717/peerj.10785 PMC7868070

[70] Aslam MM, Deng L, Meng J, et al. Characterization and expression analysis of basic leucine zipper (bZIP) transcription factors responsive to chilling injury in peach fruit. Mol Biol Rep. 2023;50:361–76. 36334232 10.1007/s11033-022-08035-3

[71] Liu H, Tang X, Zhang N, et al. Role of bZIP transcription factors in plant salt stress. Int J Mol Sci. 2023;24:7893. 37175598 10.3390/ijms24097893 PMC10177800

[72] Hartmann L, Pedrotti L, Weiste C, et al. Crosstalk between two bZIP signaling pathways orchestrates salt-induced metabolic reprogramming in Arabidopsis roots. Plant Cell. 2015;27:2244–60. 26276836 10.1105/tpc.15.00163 PMC4568499

[73] Jia D, Shen F, Wang Y, et al. Apple fruit acidity is genetically diversified by natural variations in three hierarchical epistatic genes: MdSAUR37, MdPP2CH and MdALMTI. Plant J. 2018;95:427–43. 29750477 10.1111/tpj.13957

[74] Li S, Liu X, Xie X, et al. CrMYB73, a PH-like gene, contributes to citric acidaccumulation in citrus fruit. Sci Hort. 2015;197:212–7.

[75] Zhao X, Fan Y, Xiang M, et al. DdaCrz1, a C2H2-Type transcription factor, regulates growth, conidiation, and stress resistance in the nematode-trapping fungus *drechslerella dactyloides*. J Fungi. 2022;8:750. 10.3390/jof8070750 PMC9322116 35887505

[76] Zhang B, Liu J, Yang ZE, et al. Genome-wide analysis of GRAS transcription factor gene family in *Gossypium hirsutum* L. BMC Genomics. 2018;19:1–12. 29743013 10.1186/s12864-018-4722-x PMC5944045

[77] Petkova T, Doykina P, Alexieva I et al. Characterization of fruit sorbet matrices with added value from *Zizyphus jujuba* and *Stevia rebaudiana*. Foods. 2022;11. 10.3390/foods11182748 PMC9498022 36140880

[78] Sun M, Liu J, Li J, et al. Endophytic bacterium *Serratia Plymuthica* from Chinese leek suppressed apple ring rot on postharvest apple fruit. Front Microbiol. 2021;12:802887. 35310399 10.3389/fmicb.2021.802887 PMC8929176

[79] Gambino G, Perrone I,Gribaudo I. A Rapid and effective method for RNA extraction from different tissues of grapevine and other woody plants. Phytochem Anal. 2008;19:520–5. 18618437 10.1002/pca.1078

[80] Wu S, Sun W, Xu Z et al. The genome sequence of star fruit (*Averrhoa carambola*). Hortic Res. 2020;7. 10.1038/s41438-020-0307-3 PMC7261771 32528707

[81] Chen C, Chen H, Zhang Y et al. TBtools: an integrative toolkit developed for interactive analyses of big biological data. Mol Plant. 2020;13. 10.1016/j.molp.2020.06.009 32585190

[82] Li XP, Zhu YT, Zhao YM, et al. Selection and validation of reference genes of *Averrhoa carambola* by quantitative real-time PCR (in Chinese). Mol Plant Breed. 2022;22:789–79.

