# Supplementary figures and images for: Identification of key gene networks controlling organic acid and sugar metabolism during star fruit (Averrhoa carambola) development
Source: BMC Plant Biol. 2024 Oct 10;24:943. doi: 10.1186/s12870-024-05621-4 (PMC11465491; doi:10.1186/s12870-024-05621-4)

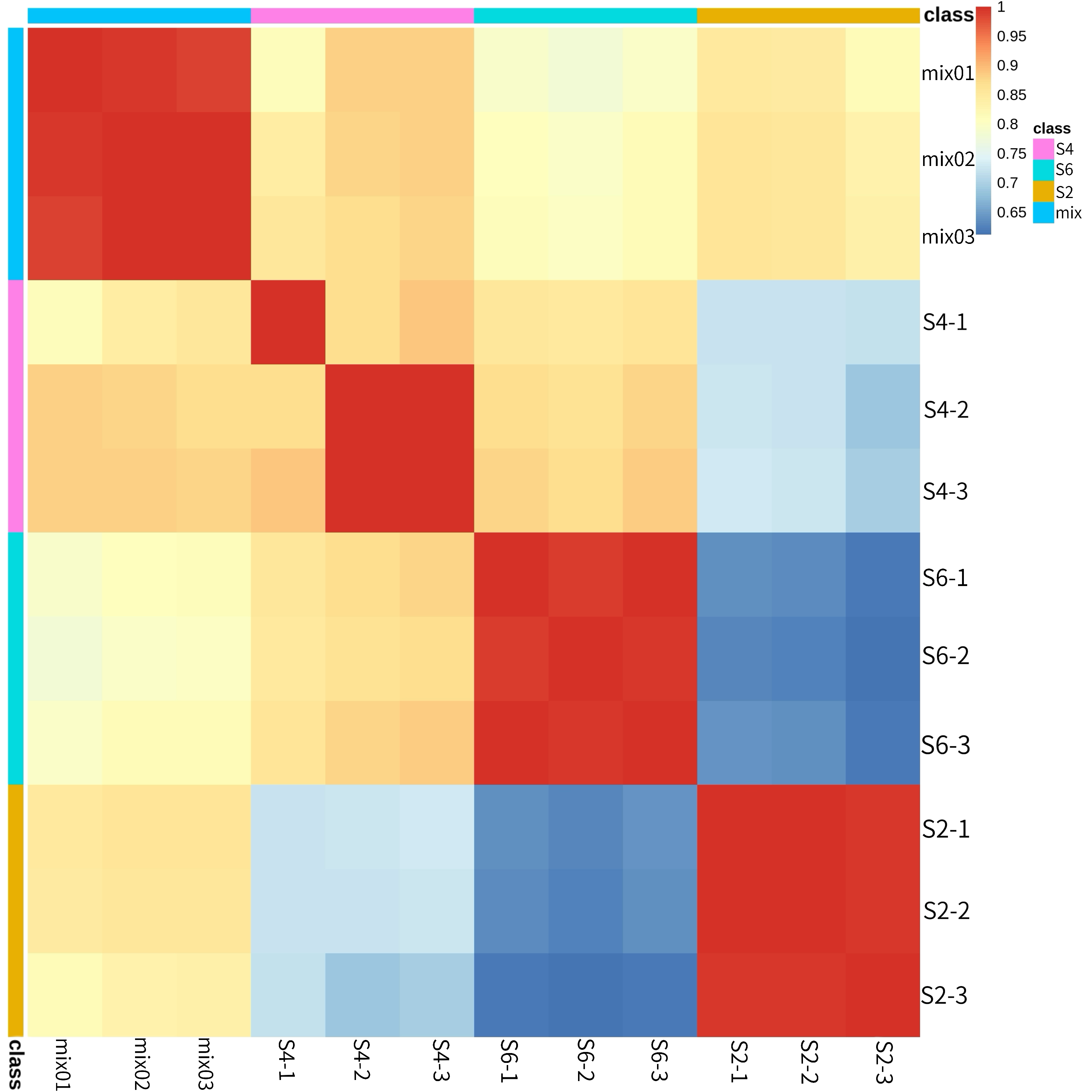

Supplement: Supplementary file 1 — Supplementary Material 1 [file 12870_2024_5621_MOESM1_ESM.png]

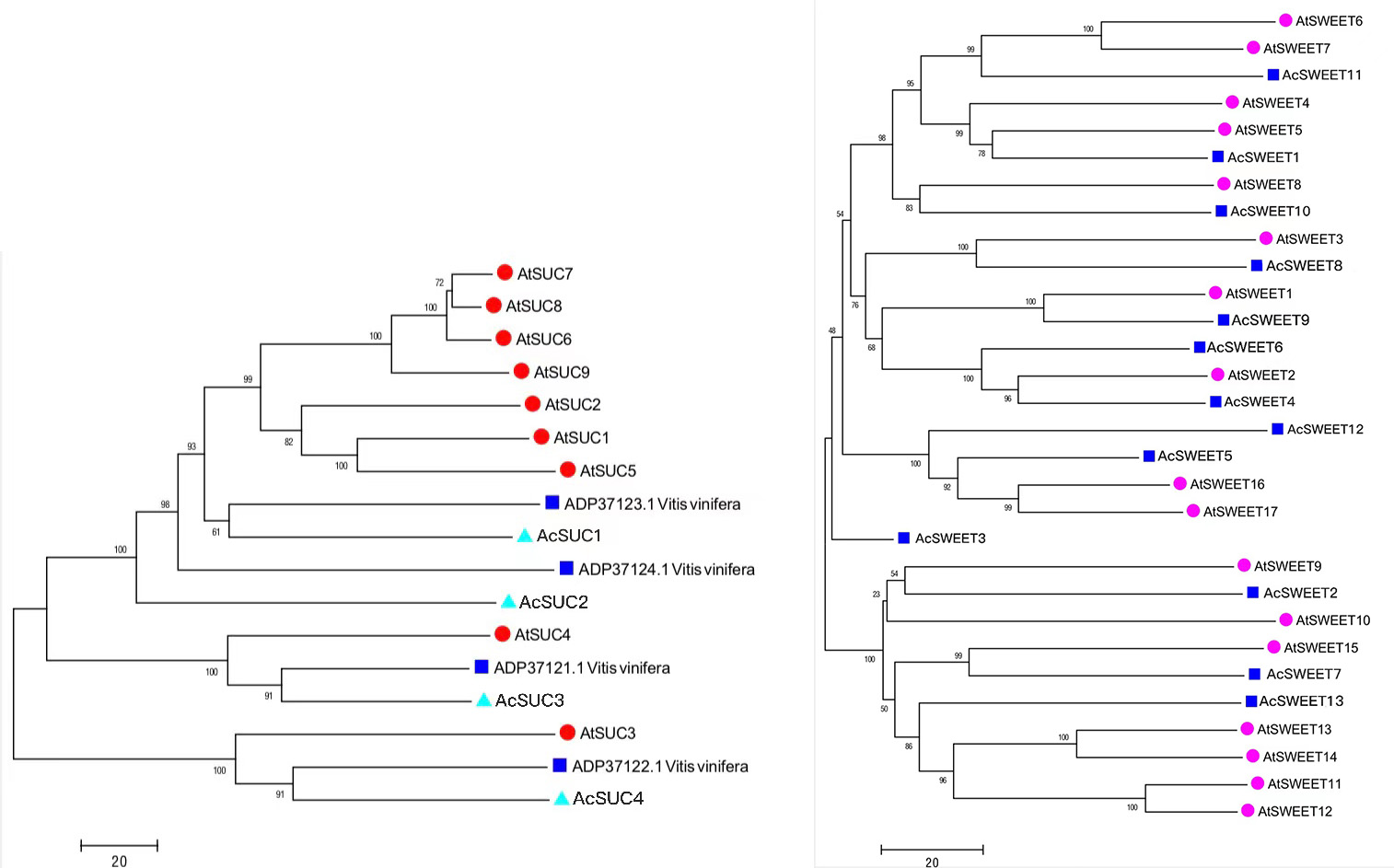

Supplement: Supplementary file 2 — Supplementary Material 2 [file 12870_2024_5621_MOESM2_ESM.jpg]

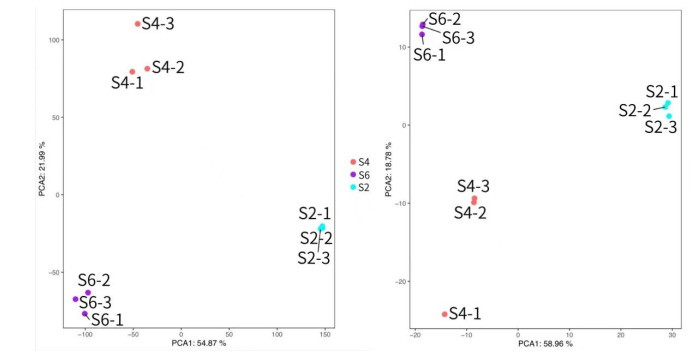

Supplement: Supplementary file 3 — Supplementary Material 3 [file 12870_2024_5621_MOESM3_ESM.jpg]

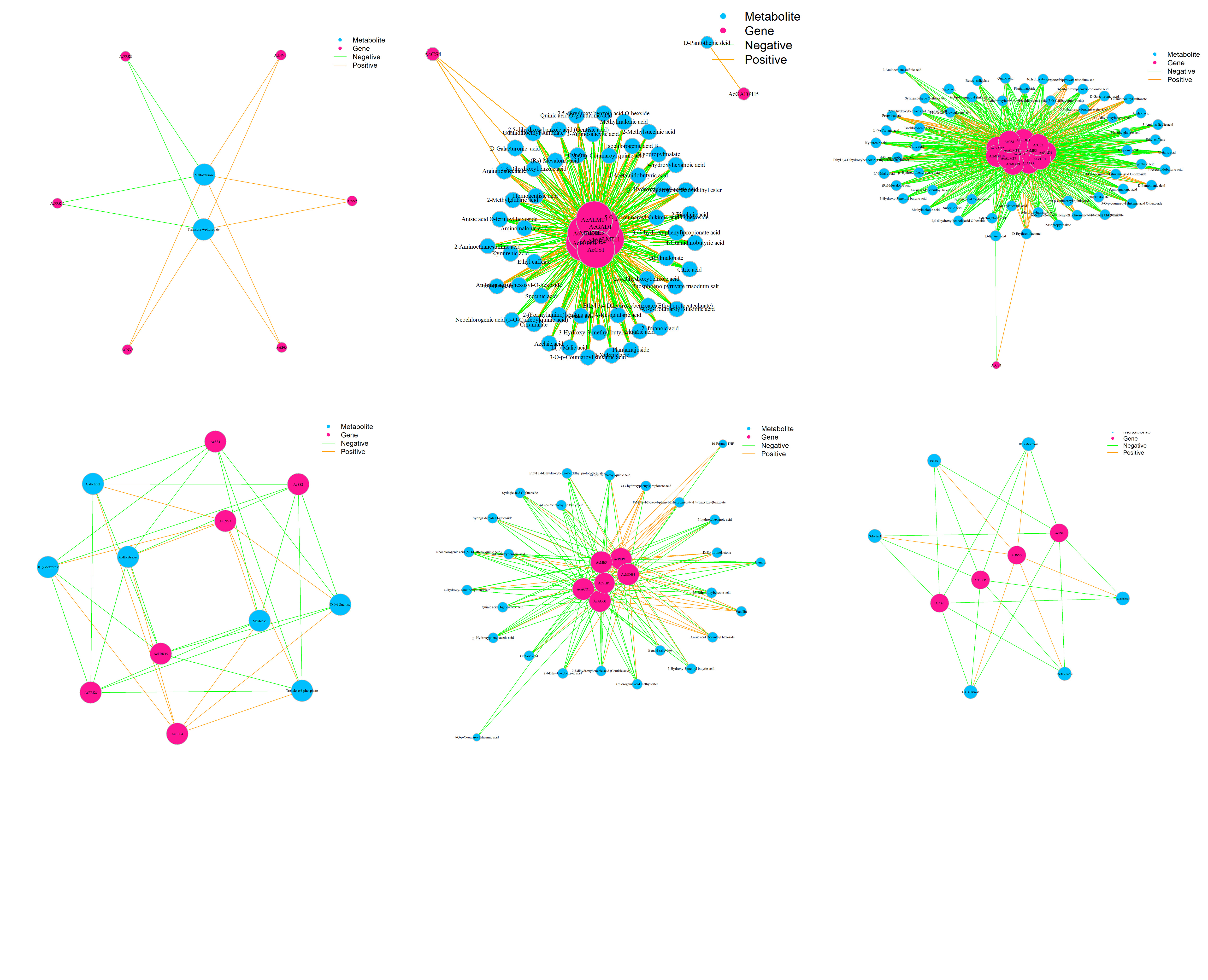

Supplement: Supplementary file 4 — Supplementary Material 4 [file 12870_2024_5621_MOESM4_ESM.jpg]
