# Supplementary material for: Identification of key gene networks controlling organic acid and sugar metabolism during star fruit (Averrhoa carambola) development
Source: BMC Plant Biol. 2024 Oct 10;24:943. doi: 10.1186/s12870-024-05621-4 (PMC11465491; doi:10.1186/s12870-024-05621-4)
Supplement: Supplementary file 6 — Supplementary Material 6 [file 12870_2024_5621_MOESM6_ESM.docx]

**Figure S1.** Correlation analysis between repeat samples.

**Figure S2.** Maximum likelihood phylogeny of sucrose synthase. Red, Purple: Arabidopsis; Mazarine: *Vitis vinifera*; Blue: *Averrhoa carambola.*

**Figure S3.** Principal component analysis of gene and metabolite

**Figure S4.** Transcription-metabolic co-expression network diagram of sugar and organic acid metabolic pathway,
